# Out of Africa: characterizing the natural variation in dynamic photosynthetic traits in a diverse population of African rice (*Oryza glaberrima*)

**DOI:** 10.1093/jxb/erab459

**Published:** 2021-10-17

**Authors:** Sophie B Cowling, Pracha Treeintong, John Ferguson, Hamidreza Soltani, Ranjan Swarup, Sean Mayes, Erik H Murchie

**Affiliations:** 1 Division of Plant and Crop Science, School of Biosciences, University of Nottingham, Sutton Bonington Campus, Loughborough, UK; 2 Advanced Data Analysis Centre, University of Nottingham, Sutton Bonington Campus, Loughborough, UK; 3 Department of Plant Sciences, University of Cambridge, Cambridge, UK; 4 University of Essex, UK

**Keywords:** African rice, dynamic modelling, natural variation, NPQ, *O. glaberrima*, photosynthesis, rice, stomatal conductance

## Abstract

African rice (*Oryza glaberrima)* has adapted to challenging environments and is a promising source of genetic variation. We analysed dynamics of photosynthesis and morphology in a reference set of 155 *O. glaberrima* accessions. Plants were grown in an agronomy glasshouse to late tillering stage. Photosynthesis induction from darkness and the decrease in low light was measured by gas exchange and chlorophyll fluorescence along with root and shoot biomass, stomatal density, and leaf area. Steady-state and kinetic responses were modelled. We describe extensive natural variation in *O. glaberrima* for steady-state, induction, and reduction responses of photosynthesis that has value for gene discovery and crop improvement. Principal component analyses indicated key clusters of plant biomass, kinetics of photosynthesis (CO_2_ assimilation, *A*), and photoprotection induction and reduction (measured by non-photochemical quenching, NPQ), consistent with diverse adaptation. Accessions also clustered according to countries with differing water availability, stomatal conductance (*g*_s_), *A*, and NPQ, indicating that dynamic photosynthesis has adaptive value in *O. glaberrima*. Kinetics of NPQ, *A*, and *g*_s_ showed high correlation with biomass and leaf area. We conclude that dynamic photosynthetic traits and NPQ are important within *O. glaberrima*, and we highlight NPQ kinetics and NPQ under low light.

## Teaser

*Oryza glaberrima*has potential adaptive traits to diverse African environments. Here we show variation in dynamic traits of photosynthesis, photoprotection, and stomatal conductance in a large accession collection.

## Introduction

The climate crisis places crop yields under increasing pressure from biotic and abiotic constraints and constitutes a major threat in meeting global food demand (Ray *et al.*, 2019). Substantial yield decreases in key cereal crops are predicted to occur in both vulnerable and productive regions (Black *et al.*, 2008; Challinor *et al.*, 2014). Asian rice (*Oryza sativa*), a dietary stable to a third of the global population, is predicted to experience yield losses up to 37% by the end of the century due to climate change-driven drought events Bocco *et al.*, 2012; Zhao *et al.*, 2017). The development of productive and resilient rice cultivars has been the subject of increasing research focus (Atwell *et al.*, 2014), and advances have been made through traditional plant breeding methods within the *O. sativa indica* and *japonica* types. However, there has also been an interest in the introgression of genes from a range of diverse interspecific material. This includes the African rice species *Oryza glaberrima*, which was domesticated in Africa 2000–3000 years ago, independently of the domestication of Asian rice *O. sativa*. *Oryza glaberrima* retains many properties that are specific to challenging African conditions of soil and climate, including limited water availability, abiotic stress, pests, and diseases (Bimpong *et al.*, 2011; Agnoun *et al.*, 2012; Bocco *et al.*, 2012; Cubry *et al.*, 2020).

**Fig. 1.**
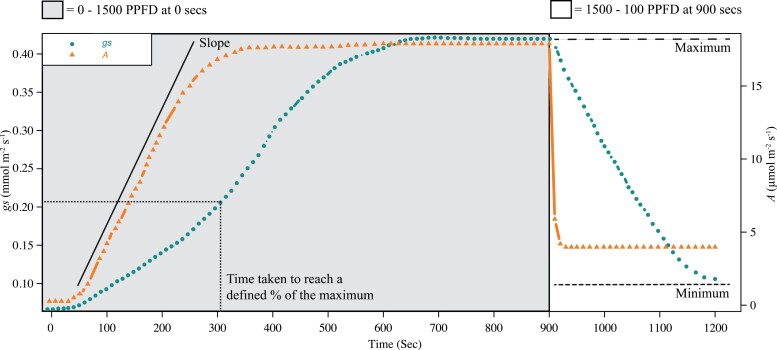
Schematic showing example induction and reduction in response to changes in light intensity during gas exchange measurements. These examples of raw *A* and *g*_s_ gas exchange measurements show the modelled dynamic response parameters; minimum, maximum, slope, and time to reach a defined percentage of the curve maximum.

*Oryza glaberrima* is not suitable for commercial rice production due to lodging, shattering, milling difficulties, and low yields in comparison with *O. sativa* (Linares, 2002). However, the resilience to a range of abiotic and biotic stresses makes *O. glaberrima* an attractive target for gene mining and translation (Fig. 1; Sarla and Swamy, 2005), which was one of the motivations for the interspecific New Rice for Africa (NERICA) breeding programme (Wambugu *et al.*, 2019). This underlying genetic diversity might allow commercial rice to tolerate increasingly unpredictable climatic conditions. Recent genomic sequencing advances for *O. glaberrima* have now added new possibilities (Cubry *et al.*, 2020).

Photosynthetic efficiency and water use efficiency (WUE) are important components of productivity and abiotic stress resilience (Zhao *et al.*, 2017). Stomata are key players in both processes, regulating CO_2_ assimilation (*A*; the parameter abbreviation list can be found in Supplementary Table S1 and the water lost by transpiration via stomatal conductance (*g*_*s*_). However, improvements in WUE incorporate a trade-off between transpiration rate at the expense of net CO_2_ assimilation rate (*A*) (Blum, 2009; Lawson *et al.*, 2010; Lawson and Blatt, 2014). The leaf stomatal density (SD) value can affect *g*_s_; recent work using rice with reduced SD has demonstrated that photosynthesis was not compromised in well-watered conditions but enhanced WUE in all conditions and improved biomass and yield under water limitation (Caine *et al.*, 2019; Mohammed *et al.*, 2019). Consequently, improved yield in water-limiting environments might be achieved by optimization of stomatal morphology and density. Dynamics of stomatal aperture alteration have also been increasingly highlighted as playing an essential role in improving photosynthetic efficiency and WUE (Drake *et al.*, 2013; Lawson and Blatt, 2014). Stomata can take some time to reach stable *g*_s_ (McAusland *et al.*, 2016). Increasing the speed of stomatal opening and closing, closely coupling to *A* (Fig. 1), may be important in conserving water and improving crop yields (Lawson and Vialet-Chabrand, 2019).

Historically, light-saturated carbon assimilation capacity (*A*_max_) (mostly under ambient atmospheric [CO_2_]) has been a parameter of interest for photosynthesis improvements (Murchie *et al.*, 2018). However, recent research now makes it clear that the dynamic responses of photosynthesis and photoprotection [such as non-photochemical quenching (NPQ)] to the fluctuating field environment are essential for photosynthetic efficiency-based yield gains (Kromdijk *et al.*, 2016; Taylor and Long, 2017; Murchie *et al.*, 2018; Acevedo-Siaca *et al.*, 2020). Light in plant canopies is transient due to architecture, intermittent cloud cover, solar angle, and wind (Burgess *et al.*, 2016). The ability of *A* to rapidly adjust to changes in light levels is limited by two major processes: stomatal dynamics and photosynthetic biochemistry (McAusland *et al.*, 2016; Slattery *et al.*, 2018; Acevedo-Siaca *et al.*, 2020). In wheat, slow induction dynamics were estimated to cost 21% of carbon assimilation acquisition (Taylor and Long, 2017). Further dynamic leaf photosynthetic efficiency can be improved through the rapid relaxation of photoprotection (Kromdijk *et al.*, 2016; Hubbart *et al.*, 2018). Under high light, NPQ dissipates excess excitation energy as heat. However, in fluctuating light conditions, NPQ dynamics can lag behind shifts in light level, limiting photosynthesis. On this basis, it is clear that elucidating photosynthesis-related dynamics is an essential focus of improving crop yields and improving abiotic stress tolerance, whereby plants can utilize light and CO_2_ with increased efficiency.

Variation in photosynthetic, NPQ, and stomatal traits have been examined in *O. sativa*; however, there is no comprehensive analysis which compares both induction and decline. We hypothesize that due to the origins within the diverse African climates, substantial variation for dynamic photosynthesis traits may exist within the genome of *O. glaberrima* and we have used a new, whole-genome re-sequenced, resource of 155 *O. glaberrima* accessions (Wambugu *et al.*, 2019; Cubry *et al.*, 2020) to characterize 58 phenotypic traits for photosynthesis and leaf WUE. This includes the use of automated machine learning to describe SD and gas exchange methods to facilitate the modelling of *A*, NPQ, and *g*_s_ induction and decline dynamics across a large population of individuals. Furthermore, as the effect of environment-driven trait adaptation is central to the novelty of *O. glaberrima*, we explore the effect of 20 climatic variables and ecotype upon trait adaptation within the population. Here, we describe an African rice population with broad heritable variation in a range of useful traits and we provide evidence that dynamic and steady-state photosynthesis and photoprotective traits are linked to whole-plant growth. To our knowledge, this is the largest survey of dynamic photosynthesis for a species in the *Oryza* genus to date. This further highlights the importance of *O. glaberrima* as an essential source of variation for crop improvement and providing a solid base for future research to elucidate physiological processes and pursue trait-related gene identification.

## Materials and methods

### Plant material and growth conditions

The seeds of 155 *O. glaberrima* accessions were provided by the Interspecies Comparison & Evolution (RICE) team within Diversité Adaptation Developpement des plantes (DIADE), IRD-Montpellier, France. A table of information presenting the plant material is provided in Supplementary Table S2.

Plants were grown, measured, and processed at the Sutton Bonington Campus, University of Nottingham, UK. Plants were sown and grown in a controlled-environment agronomy-style glasshouse (Cambridge HOK, UK). Conditions were maintained at a 12 h dark:light (07.00–19.00 h) photoperiod, controlled using blackout blinds, temperature of 28±3 °C, and 50–60% relative humidity. Metal halide lamps were used to maintain light levels when they fell below 200 μmol m^−2^ s^−1^ photosynthetically active radiation (PAR). Seeds were heat treated to prevent pathogenesis at the primary seedling stage through immersing in water at 55 °C for 15 min. Seeds were germinated in a module tray for 2 weeks before being transplanted to soil pits (5 m×5 m×1.25 m, L×W×D) within the glasshouse. Five replicates of each accession were transplanted in east–west rows, at 20 cm intervals, into high nutrient loam-based soil in 2×5 m concrete pits. Plants were irrigated by drip tapes twice a day, to provide a soil water content close to field capacity. Soil top layers were replaced every 2 weeks from the same batch.

Due to the size of the population accessions, planting was staggered at 1–2 week intervals. Accessions were grown in rotations of 12 genotypes at a time, with five biological replicates, four of which were selected for measurement. Plants were measured at 8 weeks old when they were approximately in the mid to late tillering stage. Measurements commenced in July 2017 and ended in October 2017. The elite *O. sativa* variety ‘IR64’ was used as a reference genotype and planted as a row in every batch (see ‘Data analysis’ below).

### Gas exchange measurements

An IRGA (infra-red gas analyser; Li-Cor 6400XT, Lincoln, NE, USA) was used on the uppermost fully expanded leaf. A light induction programme was used: leaves were dark adapted for 1 h, the sample leaf was then placed in the leaf cuvette and allowed to achieve steady state in darkness before being subject to a photosynthetic photon flux density (PPFD) of 1500 µmol m^−2^ s^−1^, from in-built red and (10%) blue LED lights, from 0 s to 900 s, reducing to 100 µmol m^−2^ s^−1^ from 900 s to 1200 s. A graphical representation of the induction assay can be seen in Fig. 1. The leaf cuvette conditions were maintained at a block temperature of 30 °C, 400 μmol^−1^ mol^−1^ CO_2_, flow rate 500 ml min^−1^, and 50–65% humidity. Gas exchange data were logged every 10 s. Measurements were collected between 09.00 h and 16.00 h. Chlorophyll fluorescence parameters were collected simultaneously, by applying a single saturating pulse before the application of actinic light to attain *F*_o_ and *F*_m_ and then at intervals of 60 s following this for the calculation of ϕPSII (PSII operating efficiency in the light), qP (photochemical quenching), and NPQ (non-photochemical quenching: measurement of a photoprotective process that estimates the rate constant for PSII heat loss) (Murchie and Lawson, 2013). Intrinsic water use efficiency (iWUE) was calculated post-data collection as CO_2_ assimilation rate (*A*)/stomatal conductance (*g*_***s***_). We calculate that vapour pressure deficit (VPD) in the cuvette was ~1.51–2.10 kPa. Saturation or near-saturation was achieved within this time scale. Raw data for *A*, *g*_s_, and NPQ are shown graphically in Supplementary Fig. S1 as individual replicates and means per accession. 

### Stomatal density and automated stomatal counting

Stomatal impressions were taken from the same area of the first fully expanded leaf where the IRGA measurements were obtained. A ~1 cm^2^ negative impression of the abaxial (basal) and adaxial (upper) leaf surface was taken using fast-drying clear nail polish and adhered to a microscope slide. Impressions were obtained after all other measurements had been taken.

Images were obtained on a Leica DM5000B light microscope at ×40 objective with 10 fields of view per impression. Due to the volume of images (13 110), a bespoke machine learning-based software was created to automatically calculate the number of stomata in each image. The software can reliably identify *O. glaberrima* and *O. sativa* stomata, showing a high correlation (*r*=0.94; *n*=540 images per counting method) between software and manual stomatal counts. Our method was based on transfer learning for deep neural networks: we have utilized a pre-trained deep model for the different datasets and adapt it for user-annotated rice stomata samples. Based on the transfer learning approach, we utilize a pre-trained object detection model trained on the standard COCO datasets (Lin *et al.*, 2014). Since our goal is detecting and classifying stomata, we use the Faster R-CNN model (Ren *et al.*, 2015) as one of the state-of-the-art methods based on deep neural networks. We used the Faster R-CNN model available in Tensorflow with the Inception-V2 architecture (Szegedy *et al.*, 2016) as the base model. Inception-V2 is a variation of Inception-V1, also referred to as GoogLeNet, which was the state-of-the-art architecture at the ImageNet competition in ILSVRC 2014. After loading the pre-trained Faster R-CNN, the last few layers of classification layers are changed to meet the aim of stomata classification and detection. In the next step, the Faster R-CNN with stomata images are trained with different hyperparameters such as learning rate and number of epochs to find out the best parameters to reduce execution time and errors. Further information on our methodology is located in File S1 at the Zenodo repository; Murchie, 2021).

### Morphological traits

Plant height, leaf area, and root and shoot dry biomass were taken at 8 weeks post-germination, after the completion of gas exchange measurements. Each plant was dug up and care taken to preserve the root system. The shoot area was measured using a LiCor LI-3100 area meter. The root ball was soaked and carefully washed to preserve root structure, as described by York (2018). The shoot and root material were then placed in a drying oven at 70 °C for 72 h before weighing for dry biomass.

### Data analysis

All data analyses were performed using R-Studio (v. 4.0.1).

To reduce the temporal and spatial effects of measuring the accessions in batches, a linear mixed-effects model (‘lme4’ package, v.1.1-26) was used to calculate best linear unbiased predictions (BLUPs) and predicted means, considering the effects of accession, sowing date, measurement date, location within the glasshouse, and, if relevant, IRGA machine (Supplementary Fig. S2). BLUPs are commonly used to account for the random effects that accompany measuring large populations in fluctuating environments (Robinson, 1991; Merk *et al.*, 2012). The coefficients of the mixed-effects model were also used to estimate broad sense heritability (*H*^2^). All results reported here use the adjusted means data generated from the mixed-effects model. Normality was tested using the Shapiro–Wilk test. A 0.01 α value was used, as the Shapiro–Wilk test tends to report false negatives in sample sizes exceeding 50 individuals. All data for IR64 were found to be normally distributed, whereas 25 out of 57 traits in the *O. glaberrima* panel showed a deviation from a statistically normal distribution. In the results, and *O. glaberrima* descriptive statistics Table 1, non-normal traits report values for median and interquartile range (IQR), whereas normally distributed traits will report mean and standard deviation. A full breakdown of IR64 and *O. glaberrima* normality statistics, box, and distribution plots can be found in Files S2–S4 at Zenodo.

**Table 1.** The range of natural variation and broad-sense heritability (*H*^2^) within a population of diverse *O. glaberrima* accessions across dynamic and static traits

Trait	Min	Max	Mean (SD)	Median (IQR)	PGV	Sig.	*H* ^*2*^
*Steady state*							
*A*_max_	17.92	22.42	20.16 (0.87)		22.30	∗∗∗	0.11
ETR_max_	104.10	144.30	123.00 (7.60)		32.69	∗∗∗	0.22
*g*_smax_	0.26	0.46	0.34 (0.03)		58.82	∗∗∗	0.17
iWUE_max_	58.34	71.81		62.63 (3.08)	21.40	∗∗∗	0.07
NPQ_max_	1.98	2.30	2.13 (0.06)		14.88	∗∗∗	0.12
ϕPSII_max_	0.16	0.22	0.19 (0.01)		32.70	∗∗∗	0.22
qP_max_	0.43	0.51	0.47 (0.02)		18.71	∗∗∗	0.17
Trmmol_max_	4.13	5.75	4.81 (0.27)		29.84	∗∗∗	0.12
VPD_max_	1.43	1.52	1.47 (0.01)		6.38	∗∗∗	0.00
**Morphological**							
Shoot:root	3.36	9.50		5.55 (1.13)	73.22	∗∗∗	0.12
Shoot biomass	2.36	7.85		4.40 (0.89)	123.33	∗∗∗	0.14
Shoot area	279.07	1068.97		652.73 (126.27)	118.51	∗∗∗	0.16
Root biomass	0.36	1.98		0.77 (0.18)	203.75	∗∗∗	0.23
Plant height	61.79	93.86	78.77 (6.20)		40.72	∗∗∗	0.18
Adaxial SD	260.16	353.50	314.08 (18.04)		29.72	∗∗∗	0.21
Abaxial:adaxial	1.05	1.40	1.24 (0.06)		18.87	∗∗∗	0.15
Abaxial SD	324.31	435.99	388.83 (22.54)		28.72	∗∗∗	0.21
**Dynamic**							
*g*_*s*i slope_	–3.90	–1.90		–2.44 (0.38)	78.43	∗∗∗	0.14
*g*_si max_	0.35	0.49	0.42 (0.03)		31.88	∗	0.11
*g*_si min_	0.06	0.14		0.08 (0.01)	112.50	∗∗	0.12
*g*_si 10_	65.50	207.09		108.19 (29.45)	126.83	∗∗∗	0.18
*g*_si 50_	179.53	324.80		223.71 (48.40)	64.17	∗	0.11
*g*_si__90_	477.70	684.41		539.21 (48.40)	37.53	NS	0.08
g_si rate_	0.0005	0.0008		0.0006 (<0.01)	45.45	∗	0.09
*A*_i slope_	–2.42	–1.50		–1.78 (0.17)	50.84	∗∗	0.10
*A*_i max_	20.44	27.83	23.43 (1.47)		31.53	∗∗∗	0.20
*A*_i min_	–1.32	–1.09	–1.22 (0.04)		18.85	NS	0.01
*A*_i 10_	49.01	140.97		63.79 (13.96)	136.43	∗∗∗	0.15
*A*_i 50_	189.56	334.26		217.84 (24.73)	64.97	∗∗	0.12
*A*_i 90_	652.34	849.54		718.78 (52.10)	27.14	NS	0.06
*A*_i rate_	0.03	0.04	0.03 (0.002)		33.33	NS	0.05
NPQ_i slope_	–3.48	–2.32		–2.72 (0.22)	42.65	∗∗∗	0.12
NPQ_i max_	2.12	2.44	2.24 (0.05)		14.29	∗∗∗	0.08
NPQ_i 10_	19.57	28.12	23.80 (1.53)		35.75	∗∗∗	0.11
NPQ_i 50_	50.98	56.44	53.65 (1.02)		10.16	NS	0.05
NPQ_i 90_	118.70	132.30	125.58 (2.71)		10.82	NS	0.03
NPQ_i rate_	0.017	0.02	0.02 (<0.01)		15.00	∗∗	0.07
*g*_sr slope_	6.48	10.49		8.05 (0.80)	49.43	NS	0.07
*g*_sr min_	-0.30	0.02		-0.13 (0.08)	228.57	∗∗	0.11
*g*_sr max_	0.38	0.61	0.48 (0.05)		48.96	∗∗	0.15
*g*_sr 10_	908.91	956.10		922.35 (8.56)	05.11	NS	0.07
*g*_sr 50_	1061.65	1385.05	1208.21 (76.16)		26.77	∗∗∗	0.26
*g*_sr 90_	1370.52	2116.04		1640.46 (266.31)	44.56	∗∗∗	0.23
*g*_sr rate_	0.0006	0.0101		0.001 (<0.01)	339.28	∗∗∗	0.25
*A*_r slope_	-332.60	-322.20		-328.45 (2.15)	3.17	NS	0.01
*A*_r__min_	2.88	3.60	3.22 (0.11)		24.05	∗∗∗	0.11
*A*_r max_	17.09	21.72	19.22 (0.96)		22.36	NS	0.04
*A*_r 10_	901.90	902.20	902.09 (0.04)		0.03	NS	0.03
*A*_r 50_	905.30	905.70		905.45 (0.05)	0.04	NS	0.03
*A*_r 90_	910.60	911.00		910.77 (0.05)	0.04	NS	0.02
*A*_r rate_	0.79	2.36		1.51 (0.09)	103.97	∗	0.08
NPQ_r slope_	–49.71	–36.27	–41.83 (2.65)		34.35	∗∗∗	0.21
NPQ_r min_	0.56	0.66	0.61 (0.02)		23.00	∗∗∗	0.16
NPQ_r max_	1.99	2.30	2.13 (0.06)		14.55	∗∗∗	0.10
NPQ_r 10_	921.31	923.66	922.60 (0.39)		0.27	∗∗∗	0.19
NPQ_r 50_	946.63	954.76	950.23 (1.41)		0.97	∗∗∗	0.19
NPQ_r 90_	985.20	1008.16	995.50 (4.13)		02.50	∗∗∗	0.19
NPQ_r rate_	0.013	0.020	0.016 (<0.01)		43.75	∗∗∗	0.14

Normally distributed traits report the trait mean and standard deviation, whilst the median and interquartile range is given for non-normally distributed traits. PGV is the percentage of genetic variation. Sig. refers to the ANOVA test between two mixed-effects models, where the accession is present as an effect in one model and not in another. A significant result suggests that the accession genotype has an effect and therefore the trait is heritable. ∗∗∗*P*<0.0001, ∗∗*P*<0.001, ∗*P*<0.01

A bespoke Python pipeline was written to identify the data point at 95% of the maximum and extract values within the induction side of a curve (File S5 at Zenodo).

The correlation analyses were completed using a Pearson correlation coefficient in the ‘Corrplot’ package (v. 0.84), with a correlation significance threshold of *P<*0.1005.

The percentage genetic variation (PGV) was calculated as follows; [(*x*_max–_*x*_min_)/*x*)]×100. Where *x*_max_, *x*_min_, and *x*, respectively, denote the maximum, minimum, and mean values for a trait in the population (Gu *et al.*, 2014). This measure is used to quantify the genetic variation of a trait within the combined population. Values >100% signify where the range is greater than the mean and represent particularly high underlying genetic variation.

### Kinetic modelling

Dynamic modelling of *A*, NPQ, and *g*_s_ was performed using a dose–response curve-fitting method, previously used to model stomatal responses by Barratt *et al.* (2021). The ‘drc’ (v. 3.0) package was used to analyse and extract several useful parameters for both curve induction and reduction responses (Ritz *et al.*, 2015), denoted by _i_ and _r_, respectively. The measured parameters are detailed in Table 1 and include curve slope (_i/r slope_), lower limit (_i/r min_), upper limit (_i/r max_), and the time taken to reach a defined percentage of the dependent variable, in this case 10 (_i/r 10_), 50 (_i/r 50_), and 90% (_i/r 90_). A representation of these parameters on *A* and *g*_s_ response curves can be found in Fig. 1. The LL.4 (log-logistic 4-parameter) model was chosen as the best fit for both the *A* induction, and *g*_s_ induction and reduction responses. The LL.3 (log-logistic 3-parameter) model was used for NPQ induction, and the W2.4 (4-parameter Weibull2) model for NPQ relaxation. The comparison of eight different models, followed by Akaike’s information criterion analysis, was used to select the best model fit. All 155 accessions were analysed for *A* induction curves; 24 accessions were removed for *g*_s_ induction and *g*_s_ reduction curve fitting due to unusable curve measurements.

Due to the volume of data, the best fitting model was selected for the induction and reduction curve for each parameter (*g*_s_, *A*, and NPQ) and then applied to all data (e.g. for *g*_s_ induction, a LL.4 model was applied to all 155 genotypes). To ensure that the model selection process captured the variation that may occur in the population, five genotypes were randomly chosen for model selection and the consensus model was used. The estimated parameters generated from the model (min, max, ed50, and slope) were manually cross-referenced to the raw data, to ensure these outputs closely represented the raw data. To evidence the fit of the selected models to the raw data, we include a table showing the model fit of a randomly selected accession for each parameter (Supplementary Table S3). We note the large SE for *A*_r slope_, probably due to the rapid and steep drop in *A* that occurs between two data points, so we attribute less confidence in this parameter.

### Multivariate and climatic analysis

The multivariate analyses, principal component analysis (PCA), and hierarchical clustering (H-clustering) methods require a complete dataset, with no missing values. Consequently, missing phenotype data were imputed using the missMDA package (v. 1.18). PCA was performed using the FactoMineR (v. 2.4) package, and the H-clustering was performed using the HCPC method. Details of the FactoMineR package and HCPC algorithm can be found in Lê *et al.* (2008).

The PCA and H-clustering of the phenotypic dataset contained all 155 *O. glaberrima* accessions and the IR64 *O. sativa* representative. The analysis included 64 quantitative trait variables and four qualitative variables, namely narrow ecotype, broad ecotype, country of origin, and African region.

**Fig. 2.**
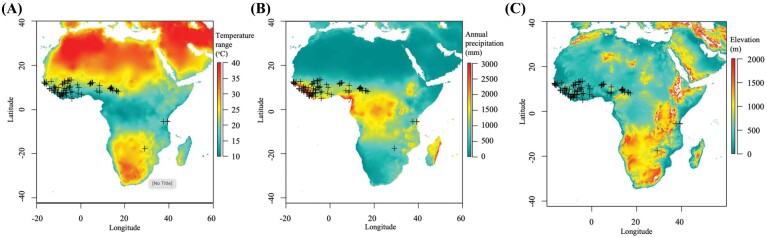
Map showing the geographical collection locations of *O. glaberrima* accessions used in this study. The annual range of (A) temperature, (B) annual precipitation, and (C) elevation across Africa.

Agroecological niche and geographical coordinates of the collection sites for each *O. glaberrima* accession were provided by AfricaRice. We have complete ecological information for all 155 accessions, and geographical coordinates for 105 accessions (Fig. 2A–C). Nineteen variables for temperature and precipitation, at the collection site of each accession, were obtained using the BIOCLIM dataset (Hijmans *et al.*, 2005). Information on the elevation above sea level was obtained using the elevatr package (v. 0.3.1). PCA and H-clustering analysis, and subsequent climate–trait correlations, were performed on the subset of 105 accessions for which we had geographical coordinates.

FactoShiny was used to generate summary reports of the PCA and H-clustering analyses on both the phenotype data and climate data; these can be found in Files S6–S9 at Zenodo.

## Results

### Phenotypic analysis of morphology and steady-state photosynthesis

Significant variation and high levels of PGV were identified between accessions across all morphological, gas exchange, and fluorescence traits measured in this *O. glaberrima* panel (Table 1; Files S2, S4 at Zenodo).

**Fig. 3.**
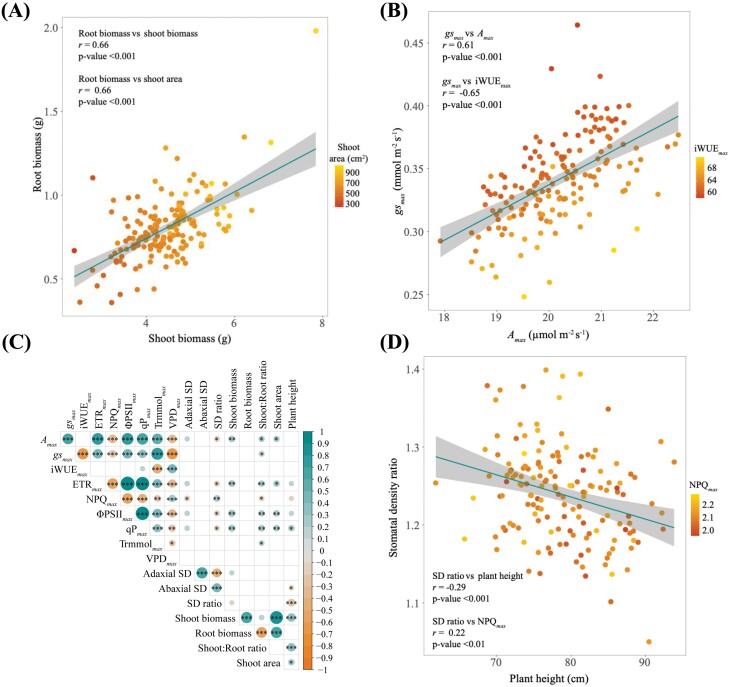
*O. glaberrima* shows a range of interesting morphological and steady-state photosynthetic trait correlations. The colour gradient shows a second correlation against the *y*-axis variable. (A) Positive correlation between root and shoot biomass; the second correlation shows root biomass and shoot area. (B) The effect of *g*_smax_ on *A*_max_ and the second correlation of *g*_smax_ against iWUE_max_. (C) Pearson correlation matrix showing associations between morphological and steady-state gas exchange traits, filtered to show trait associations at a *P*<0.1005 significance threshold. Correlations are scaled by colour, shown in the right-hand scale bar; asterisks indicate significance between traits (∗∗∗*P*<0.001, ∗∗*P*<0.01, ∗*P*<0.05). (D) Negative correlation between SD ratio and plant height, while the SD ratio against NPQ_max_ shows a positive correlation.

Root biomass, shoot biomass, and shoot area showed a 5-, 3-, and 4-fold variation, respectively. Plant height showed a 1.3-fold variation in PGV. Significant (*P*<0.001) positive correlations between all plant growth traits were found (Fig. 3A, C).

Even though key steady-state photosynthesis traits showed a relatively narrow distribution (typically between 15% and 40%), both shoot biomass and shoot area showed significant (*P*<0.01–0.05; Fig. 3A; File S10 at Zenodo), positive correlations to *A*_max_, qP_max_, ETR_max_, and ϕPSII_max_, providing confidence that steady-state photosynthesis is linked to biomass production. *g*_smax_ showed an almost 2-fold variation across *O. glaberrima* accessions. PGV for steady-state traits ranged from 6.38% to 58.82% (Table 1), with most traits in the 20–30% range, including key photosynthetic traits. All key steady-state photosynthetic traits showed significant (*P*<0.0001) positive correlations to one another (Fig. S3). Some unexpected relationships were apparent, for example between NPQ_max_ and VPD_max_. iWUE_max_ was highly correlated with *g*_smax_ (Fig. 3B) (and Trmmol_max_) but not *A*_max_, indicating stomatal limitation of *A*.

**Fig. 4.**
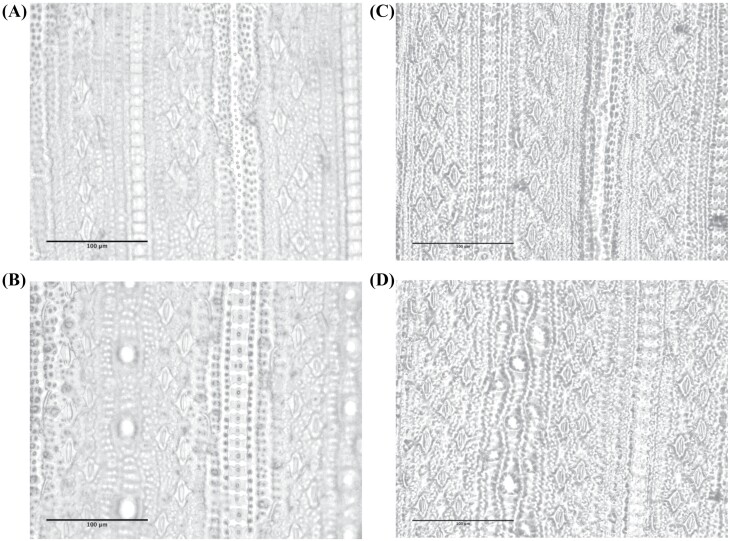
Microscope images showing examples of the *O. glaberrima* accessions with highest (TOG_14116) and lowest (TOG_5464) recorded SD. These images demonstrate the extent of SD variation in the population and the qualitative correlation between high SD and small stomatal size, (A) TOG_5464; adaxial SD=260 mm^−2^, (B) TOG_5464; abaxial SD=325 mm^−2^, (C) TOG_14116; adaxial SD=345 mm^−2^, (D) TOG_14116; abaxial SD=426 mm^−2^.

Stomatal morphology (Fig. 4) did not show a clear relationship with conductance. A relatively modest 1.3-fold accession-dependent variation in the abaxial SD and adaxial SD was observed. The abaxial SD was 1.24-fold greater than the adaxial SD. PGV showed that all SD traits were highly significant (*P*<0.0001, Table 1) across the *O. glaberrima* accessions, revealing that SD has a genetic basis. However, no association between any SD traits and iWUE_max_, or *g*_smax_ was detected. Unexpectedly the adaxial SD showed a significant negative correlation to NPQ_max_, while abaxial SD showed a negative association with plant height (Fig. 3D). The SD ratio, however, showed significant associations with multiple traits (Fig. 3C); *A*_max_, ETR_max_, ϕPSII_max_, qP_max_, NPQ_max_, and plant height, the reasons for which are unclear.

### Phenotypic analysis of dynamic photosynthesis

**Fig. 5.**
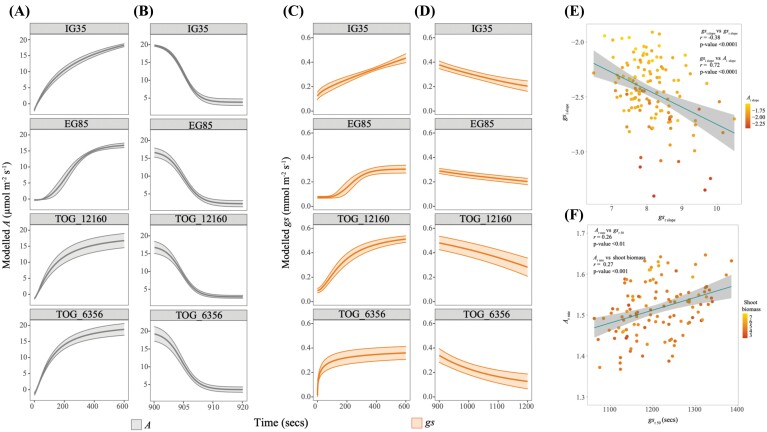
Demonstrating the variation of (A, B) *A* and (C, D) *g*_s_ dynamic responses to light intensity changes within the *O. glaberrima* population using four example accessions. IG35 and TOG_6356 were used as example of a ‘slow’ and ‘fast’ responding accession, respectively, whereas EG85 and TOG_12160 are used to demonstrate the intermediate gradient of responses in the population. (E) *g*_si slope_ was correlated with *g*_sr slope_ and *A*_i slope_; during induction, with a greater negative value indicating a steeper slope; this relationship is reversed for the decrease. (F) *A*_r rate_ shows positive associations with *g*_sr 50_ and shoot biomass.

**Fig. 6.**
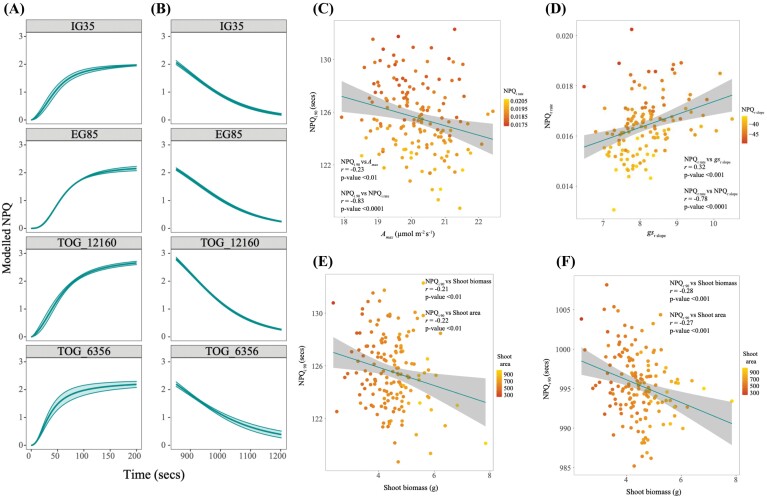
Demonstrating the variation of NPQ (A) induction and (B) relaxation responses to light intensity changes within the *O. glaberrima* population using four example accessions, as explained in Fig. 5. (C) Negative correlations were identified with NPQ_i 90_ against *A*_max_ and NPQ_i rate_. (D) NPQ_r rate_ showed a positive relationship to *g*_sr slope_, where a high value indicates a steeper slope and a negative correlation between NPQ_r rate_ and NPQ_r slope_, where for this model a more negative value suggests a steeper relaxation slope. NPQ_i 90_ (E) and NPQ_r 90_ (F) showed associations with both shoot biomass and shoot area.

Dynamic responses are now recognized as important determinants of photosynthetic productivity. Responses of gas exchange, fluorescence, and photoprotection to light shifts were modelled and show significant variation in 29 traits (Table 1, column ‘Sig’; Figs 5A–D, 6A, B; Supplementary Fig. S4A–F).

The well-documented divergence between the induction of *g*_s_ and *A* was observed, where a lag in *g*_s_ induction and reduction occurs relative to *A* (Figs 1, 5A–C). The mean upper limit estimates for *A* induction and reduction curves (*A*_i max_ and *A*_r max_) and *g*_s_ induction and reduction (*g*_sr max_ and *g*_si max_) curves were similar (*P*<0.0001, Supplementary Fig. S5A, B) to measured values. The estimated averages for the mean lower limits of the *A* induction (*A*_i min_), *g*_s_ induction and reduction (*g*_si min_ and *g*_sr min_) curves are close to zero (Table 1).

The average time taken to reach 10% of the maximum induction curve was significantly less for CO_2_ assimilation (*A*_i 10_) than for *g*_s_ (*g*_si 10_), whereas the time taken to reach 50% of the induction curve for *A*_i 50_ and *g*_si 50_ did not significantly differ. However, the average time to reach induction to 90% of the maximum (*A*_i 90_) was significantly longer than that of *g*_si 90_.

Strong interactions between stomatal and CO_2_ assimilation indicate co-dependence (Fig. 5E, F). Notably the steepness of the *g*_s_ induction slope (*g*_si slope_) highly correlates with key induction traits *g*_si 90_, *g*_si rate_, *g*_smax_, *A*_i slope_, *A*_i rate_, _i_*WUE*_max_, NPQ_islope_, NPQ_imax_, and NPQ_i50_. *g*_si rate_ was also strongly correlated to many dynamic induction traits; *g*_smax_, *g*_si 10_, *g*_si 50_, *g*_si 90_, *A*_max_, *A*_i 90_, and *A*_i rate._

The rate of photosynthetic induction in high light was associated with rates of decline in low light (Fig. 5). *g*_si slope_ versus *g*_sr slope_, *g*s_i rate_ versus *gs*_r rate_, and *A*_i rate_ versus *A*_r rate_ were significant, suggesting that accessions which exhibited rapid stomatal opening also close at a greater rate (Fig. 5C, D; Supplementary Fig. S5). Further, traits associated with rapid stomatal closure, *g*_sr slope_, *g*_sr 10_, *g*s_r 50_, and *g*_sr 90_ showed significant associations with enhanced iWUE_max_.

Like steady-state traits, *A* and *g*_s_ dynamics were also linked to plant biomass and morphology in these data, further supporting the role of photosynthesis in determining growth. A greater *A*_i rate_ was positively correlated with total plant and shoot biomass. *A*_r rate_ showed positive associations with total plant biomass, shoot biomass, shoot:root ratio, and shoot area. *A*_r slope_ had negative associations with shoot biomass, shoot:root ratio, and plant height, while a more rapid *A*_r 90_ was correlated to a greater shoot biomass, shoot:root ratio, and shoot area. *g*_si rate_ showed positive associations with total plant biomass, shoot biomass, root biomass, and shoot area.

Again there were fewer links with stomatal morphology; a significant negative association was identified between the SD ratio and *A*_r rate_. Only upper leaf SD was also found to have positive relationships to *g*_si 50_ and *A*_r rate_, and a negative relationship to *g*_sr min_.

### Non-photochemical quenching dynamics

NPQ is of particular interest here because it showed multiple relationships with photosynthesis and biomass. The model estimation of the NPQ induction and relaxation curve upper limit (NPQ_i max_ and NPQ_r max_) was close to the measured value for NPQ_max_, providing confidence in the method (Table 1; Supplementary Fig. S5C)

We observed limited significance between the kinetics of NPQ relaxation and kinetics of *A*. Importantly, there was a significant negative correlation between the *A* reduction curve lower limit (*A*_r min_) achieved under 100 PPFD, and NPQ_r slope_, NPQ_r 50_, and NPQ_r 90_, suggesting that *A* maintains a higher value under low light conditions when NPQ relaxes rapidly (Kromdijk *et al.*, 2016). Additionally NPQ_i slope_, NPQ_i rate_, and the time taken to induce 90% of the maximum (NPQ_i 90_) strongly correlated with *A*_max_.

Speed of induction was not closely related to NPQ capacity: only the time taken to reach 90% of the NPQ curve upper limit (NPQ_i 90_) positively correlated to a greater NPQ_max_. Like gas exchange traits, NPQ induction and relaxation traits were positively correlated (NPQ_i slope_ versus NPQ_r slope_ and NPQ_i rate_ versus NPQ_r rate_).

Interestingly, NPQ and *g*_s_ dynamic traits also showed numerous significant correlations. *g*_si slope_ significantly correlated with NPQ_i slope_, NPQ_i 10_, NPQ_i 50_, and NPQ_i 90_. *g*_si rate_ was positively related to NPQ_i slope_ and NPQ_i 90_. Accessions with steeper *g*_sr slope_ were also found to have a greater NPQ_r rate_ (Fig. 6D). These associations highlight a complex interdependent relationship between *g*_s_, *A*, and NPQ and the recent link noted between underlying control of NPQ by PsbS and the dynamics of stomatal conductance and gas exchange (Fig. 6C, D) (Kromdjik *et al*., 2016; Glowacka *et al.*, 2018).

Further NPQ relaxation traits were related to morphological and SD traits, indicating that photoprotection has a role in determining growth. NPQ_i slope_ and NPQ_i 90_ (Fig. 6E) showed negative correlations with shoot biomass and shoot area. NPQ_i rate_ positively correlated to shoot biomass and shoot area. A more pronounced set of associations was observed during NPQ relaxation; shoot biomass and shoot area, respectively, showed negative correlations to NPQ_r slope_, NPQ_r 50_, and NPQ_r 90_ (Fig. 6F), and positive correlations to NPQ_r 10_ and NPQ_r rate_. Root biomass showed a similar, but not as strong, association with NPQ_r slope_, NPQ_r 10_, NPQ_r 90_, and NPQ_r rate_.

### Trait and ecological comparison between *O. glaberrima* and *O. sativa*

It is informative to compare the *O. glaberrima* trait variation with that of the elite Asian *O. sativa* cultivar, IR64 (File S3 at Zenodo; Table 1) even though caution should be observed using just one genotype. We highlight the slower induction rates of photosynthesis of IR64.

IR64 had a slightly smaller shoot than *O. glaberrima* but a greater root biomass, reflected in the lower shoot:root ratio of IR64, suggesting a greater investment in roots. IR64 height was lower. IR64 displayed a greater SD on the abaxial leaf side than *O. glaberrima*, and IR64 had a lower SD ratio.

IR64 did not differ from *O. glaberrima* for *A*_max_ and NPQ_max_. However, average ETR_max_ and ϕPSII_max_ were higher in IR64. IR64 showed a slightly lower *g*_smax_ and greater iWUE_max_ in comparison with *O. glaberrima*. The latter is likely to be a direct result of the higher levels of *g*_smax_ observed in *O. glaberrima*. Clear differences were found in dynamics of *A*, *g*_s_, and NPQ between the two species.

During induction, IR64 was significantly slower than *O. glaberrima* for *g*_si 10_, *g*_si 50_, *A*_i 10_, *A*_i 50_, *A*_i 90_, *A*_i rate_, NPQ_i 10_, and NPQ_i 50_ (Supplementary Fig. S4). This implies that IR64 had a longer *g*_s_ and NPQ lag phase. The initial rapidity of the *g*_s_ induction curve may facilitate the significantly faster *A* response observed in *O. glaberrima*, suggesting that *O. glaberrima* may be able to respond better to the onset of high light than IR64. During the decrease, IR64 and *O. glaberrima* did not significantly differ for *g*_sr 10_, *g*_sr 50_, *g*_sr 90_, g_sr rate_, *A*_r 10_, *A*_r 90_, *A*_r rate_, NPQ_r 10_, and NPQ_r rate_. IR64 was found to have a faster reduction response for *A*_r 50_, NPQ_r 50_, and NPQ_r 90_ in comparison with *O. glaberrima* (Table 1 in comparison with File S3 at Zenodo).

During the multivariate analyses, we observed that *O. sativa* IR64 values cluster separately from *O. glaberrima* for both ecology and country of origin. This can be seen most clearly when plotting principal components (PCs) 1 and 3, where the two species cluster distinctly for the Asian country of origin and paddy field ecology (Fig. 8C).

### Impact of country of origin and ecotype on *O. glaberrima* trait adaptation

An important aspect of *O. glaberrima*’s novelty is the independent evolution to *O. sativa* and adaptation to the variable African environment. We used PCA and H-clustering to explore natural trait variation and the adaptive effect of environmental climatic variables. Here we identify phenotypic trends which cluster according to country and environment, indicating adaptation and possibly variation in growth strategy.

**Fig. 7.**
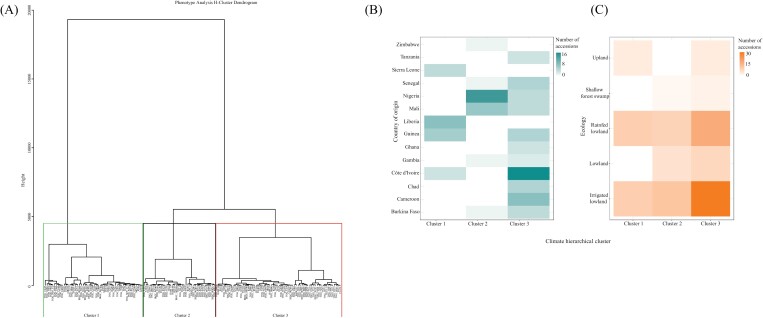
Hierarchical clustering of 155 *O. glaberrima* accessions (A) for 64 phenotypic traits and the frequency of accessions for each country of origin (B) and ecological niche (C) in the clades (1–3) identified in the climate hierarchical clustering analysis.

The PCA and H-clustering were separated into two grouped analyses for phenotypic and climatic variables. For the PCA of phenotypic traits 12 PCs were selected as they explain 95% of the variance (Supplementary Fig. S6). The H-clustering analysis identified three clusters (Fig. 7A) with common sources of trait variation (File S7 at Zenodo). The accessions in cluster 1 are characterized by a slow *g*_s_ reduction time (*g*_sr 10/50/90_), rapid *A* and NPQ induction time (*A*_i 50_ and NPQ_i 90_), steep *A* reduction curve (*A*_r slope_), rapid *A* reduction time (*A*_r 50/90_), high values for *g*_smax_, *A*_r rate_, NPQ_i rate_, and shoot:root ratio, and low values for root biomass, VPD_max_, and iWUE_max_. Accessions present in cluster 2 demonstrate *g*_s_ reduction curves with a steep slope and rapid reduction times (*g*_sr 10/50/90_), high trait values for NPQ_max_, VPD_max_, and iWUE_max_, and low values for plant biomass, shoot biomass, shoot area, *g*_smax_, ETR_max_, and ϕPSII_max_. Accessions in the largest group, cluster 3, show high trait values for total biomass, shoot biomass, shoot area, root biomass, and *A*_max_, low levels of NPQ (NPQ_r min_) under reduced light (100 PPFD), and rapid *g*_s_ reduction time (*g*_sr 50/90_). Cluster 3 is the group where IR64 can be found, and it consists mostly of lowland type accessions.

**Fig. 8.**
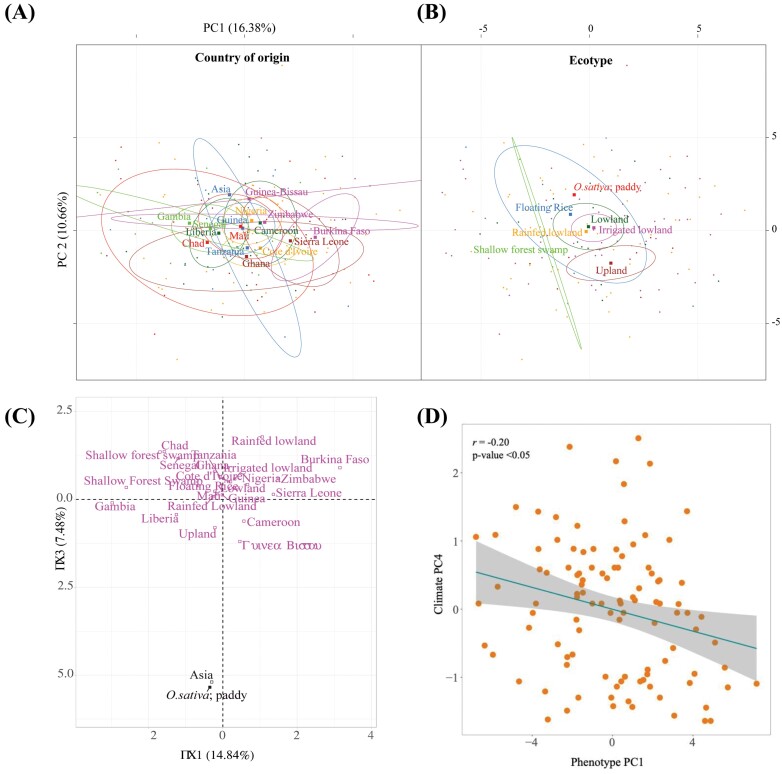
Graphical PCA outputs; the phenotypic PCs 1 and 2 are overlaid with 95% confidence ellipses for the *O. glaberima* accessions; (A) country of origin and (B) ecotype categorical variables. (C) PCs and 3 show the separate clustering of *O. sativa* (IR64), based on country of origin and ecotype categories, from *O. glaberrima.* (D) PC1 from the phenotypic traits data PCA was found to be a function of PC4 from the climatic data PCA analysis.

Adaptation to different environments was explored during the multivariate analyses. In Fig. 8A and B, axes PC1 and 2 are shown overlaid with ecological niche and country of origin. *Oryza glaberrima* accessions cluster separately dependent upon their ecological origin, in particular upland or lowland (Fig. 8B). Accessions from lowland-type ecologies dominate, though it is still clear that upland and lowland show trait differences. Accessions also show a high degree of trait variation due to countries of origin that have contrasting climates (Fig. 8A). For example, distinct clustering can be seen between landlocked Burkina-Faso, which borders the Sahara, and coastal Gambia. A categorical analysis was performed to establish if the accessions that occupy each cluster of the H-clustering analysis share similar origins (Supplementary Fig. S7B, C). While there is no obvious relationship, a greater proportion of upland accessions occupy cluster 1, whereas a large proportion of lowland accessions are present in cluster 3 (Supplementary Fig. S7C).

The diversity of climates and elevations (Fig. 2A–C) are likely to have directly impacted trait adaptation and resilience. A PCA focused on climatic traits explored the relationship between climate and phenotype. The first four PCs explain 90% of trait variation in the population (File S8 at Zenodo). H-clustering identified three distinct clusters of accessions with common sources of variation in climatic variables (Files S9, S11 at Zenodo). A categorical analysis of ecological niche and country of origin for the accessions present in each cluster showed a clear distinction of climate-based clustering due to country of origin (Fig. 7C). Cluster 1 contains all accessions that originate from the neighbouring countries of Liberia and Sierra Leone. Cluster 2 contains all accessions from Zimbabwe and most accessions originating from Nigeria. Cluster 3, which contains the largest number of accessions, contains all individuals originating from Cameroon, Chad, Ghana, and Tanzania, and the majority of accessions from Côte d’Ivoire and Senegal.

With the extensive phenotypic and climatic variables reduced to a small number of components, we completed a correlation analysis between the phenotypic and climatic trait PCs to identify groups of climatic drivers on trait adaptation. A significant positive association was identified between trait PC1 and climatic PC4 (*r*= –0.20, *P*<0.05; Fig. 8D), suggesting that key traits contributing to phenotypic trait PC1, which includes photosynthetic traits and shoot biomass, have adapted in response to precipitation-related variables that contribute to climate PC4 loadings. Other significant associations were identified between phenotype PC5 and climate PC4 (*r*=0.25, *P*<0.05), phenotype PC8 and climate PC2 (*r*=0.24, *P*<0.05), and phenotype PC11 and climate PC3 (*r*=0.25, *P*<0.05) File S12; Supplementary Fig. S8).

## Discussion

Crop production in future climates has the challenge of increasing productivity whilst retaining resilience. To do so, optimizing interactions and trade-offs between carbon assimilation, photoprotection, and water loss will be essential. However, we do not yet have complete understanding of the genetic basis of the co-regulation of the interlinked processes and components (light harvesting, photoprotection, electron transport, carbon assimilation, and stomatal conductance) involved. Recent progress shows that crop productivity and WUE are only partly dependent upon ‘steady-state’ maximum values of *A*_max_ and *g*_smax_. SD, stomatal conductance, and photoprotection dynamics have been identified as critical traits to optimize carbon assimilation and minimize abiotic stress (Kromdijk *et al.*, 2016; Caine *et al.*, 2019; Faralli *et al.*, 2019). However, elite gene pools may be genetically narrow and poorly adapted to challenging environmental conditions. Attention is increasingly focused upon underutilized crop species and wild relatives as a source of genetic diversity to improve resilience in commercial species (Draic *et al.*, 2011). Whilst the variation for photosynthesis induction has been partly characterized in *O. sativa*, this is not true of *O. glaberrima* (Acevedo-Siaca *et al*., 2020, 2021). The *O. glaberrima* association panel used here was developed as a resource for crop improvement, which may have diversity not available in *O. sativa* (Agnoun *et al.*, 2012). For the first time, a comprehensive analysis of photosynthesis- and morphology-related traits has been completed in *O. glaberrima*. Our novel approach uses a large pool of accessions, with a large range of heritable natural variation to explore the natural variation and relationships in these traits. While we cannot here make a meaningful comparison between *O. glaberrima* and *O. sativa*, we observed key differences, with the former showing faster photosynthesis induction. This may be an indication of adaptation to drier soils and air generally, requiring faster opening and closure of stomata (Lawson and Vialet-Chabrand, 2019).

Here we have described extensive natural variation in *O. glaberrima* for steady-state, induction, and relaxation/reduction responses for *A* and g_s_. This suggests underlying genetic diversity to these traits that could be identified and exploited. We identified indications of heritability (*H*^2^) and underlying genetic variation (PGV) in many of these traits (Table 1). Trait heritability values were comparable with estimates of similar traits from maize (Choquette *et al.*, 2019), but they are marginally lower than those previously demonstrated in *O. sativa* (Qu *et al.*, 2017), though a strong genetic component is still indicated. A calculation of heritability using genomic data would provide a more accurate estimation (Zhu and Zhou, 2020). This would be useful when selecting traits for genetic introgression or characterization. The large number of accessions used here (155) permits a statistical comparison that was not possible in related studies on dynamic photosynthesis in *O. sativa* where fewer lines were analysed (Acevedo-Siaca *et al.*, 2020).

A global PCA and clustering analysis showed a distinction between clusters of high biomass (cluster 3), low biomass (cluster 2), and low root biomass (cluster 1). The fast *g*_s_ decrease, low *g*_smax_, high NPQ_max_, and high iWUE_max_ of cluster 2 would suggest a conservative type geared toward water conservation, whilst the high total biomass of cluster 3 is consistent with a fast growth type displaying a rapid *g*_s_ decrease, low NPQ, and a higher *A*_max_. The association of cluster 3 with wetter lowland environments is consistent with higher productivity. We therefore see a general consistency in these two clusters with photosynthetic, water use, and biomass production ‘strategy’. It is also notable that steady-state *A*_max_ correlates well with biomass, suggesting that capacity for higher photosynthesis is still important. Increases in photosynthetic capacity are known to improve light responses in rice (Sun *et al.*, 2016).

### Extensive natural variation identified in dynamic photosynthetic traits

In recent years, there has been a shift in photosynthesis-related research towards dynamic responses in place of steady-state values. It is now recognized that irradiance fluctuations in field conditions, and the ability of stomatal and photosynthetic responses to respond instantaneously, can substantially affect plant productivity (Taylor and Long, 2017). To enable greater productivity in dynamic environments such as a crop canopy, one would anticipate that all components of photosynthesis would respond rapidly to ‘track’ light closely. Each component has a different effect; thus, fast activation of the Calvin cycle and CO_2_ assimilation during induction is beneficial, while rapid reduction of NPQ and fast stomatal closure at transition to low light enable the attainment of improved CO_2_ efficiency and iWUE at low light.

It is clear that we see some independence of dynamic traits, but interesting associations appear which indicate a link with biomass. Recent research suggests that major yield gains can be made by enhancing photoprotection capacity and NPQ dynamic responses (Kromdijk *et al.*, 2016; Hubbart *et al.*, 2018). Rapid NPQ relaxation can remove the limitation on quantum yield of CO_2_ assimilation, allowing a quicker recovery of photosynthetic efficiency upon *A* reduction (Kromdijk *et al.*, 2016; Murchie and Ruban, 2020). Our findings support this: NPQ relaxation dynamics were the only group found to have ubiquitous associations with increased shoot biomass and area. Notably, we also observed that values for *A* under low light were greater in those accessions that exhibited rapid NPQ relaxation and those that have lower NPQ capacity under low light (NPQ_r min_). It is also hypothesized that faster induction of CO_2_ assimilation may reduce the need for photoprotection during induction (McAusland and Murchie, 2020); however, we found no association between *A* induction traits and NPQ dynamic or steady-state values. We did find that faster NPQ induction is associated with greater photosynthetic capacity, shoot area, and biomass.

Whilst no associations were identified between NPQ and *A* reduction dynamics, we found strong positive correlations between the speed of *g*_s_ and NPQ dynamics. This may highlight the importance of the key NPQ protein, PSII subunit S (PsbS), on stomatal conductance, as shown by Głowacka *et al.* (2018), whereby PsbS overexpression, which increases both NPQ capacity and NPQ dynamic rate (Kromdijk *et al.*, 2016; Głowacka *et al.*, 2018; Hubbart *et al.*, 2018), also reduces the extent of stomatal opening in tobacco. This may be reflected here by the negative correlation between NPQ_max_ and *g*_smax_, also that the *g*_s_ induction rate was lower when NPQ induction was faster. This highlights the need to further explore the associations between NPQ and *g*_s_ dynamics: these have not been elucidated although there is a general principle that limitations imposed by *g*_s_ or Rubisco activation state would result in a further reduction of electron transport and an enhanced NPQ. We suggest that in *O. glaberrima* NPQ may be a major player in both *g*_s_ and *A* reduction dynamics. Akin to the relationship between *A* and *g*_s_, there is a trade-off in NPQ as it reduces photosynthetic quantum yields under low irradiance.

No association was identified between the water use-related traits, *g*_s_ and iWUE_max_, and SD; this may be because the variation was less than that needed to produce changes in gas exchange traits (Caine *et al.*, 2019; Mohammed *et al.*, 2019). It is also possible that this highlights the importance of stomatal size and morphology, rather than density, on these traits. Smaller stomata have been shown to have improved WUE, *g*_smax_, and dynamics (Drake *et al.*, 2013; Dittberner *et al.*, 2018; Lawson and Vialet-Chabrand, 2019; Chatterjee *et al.*, 2020). However, the positive correlations we identified between SD ratio, NPQ_max_, and the level of NPQ achieved under low light (NPQ_r min_) is unusual. The significant negative association between SD ratio and *A*_r rate_ has no direct interpretation but may indicate that the SD ratio is a trait worthy of further work. Upper leaf SD had positive relationships to *g*_si 50_ and *A*_r rate_, and negative to *g*_sr min_, also indicating that distinction between the leaf surfaces may be important.

Understanding the interplay of photoprotective, stomatal, and assimilation dynamics should include detailed morphological characterization (Ohsumi *et al.*, 2007; Drake *et al.*, 2013; McAusland *et al.*, 2016), together with the associated mesophyll conductance (Campany *et al.*, 2016; Deans *et al.*, 2019). The proportion by which photosynthetic dynamics are limited by stomata or biochemistry seems to be species dependent (Tinoco-Ojanguren and Pearcy, 1993; Taylor and Long, 2017; De Souza *et al.*, 2020). *Oryza sativa* photosynthetic induction has been shown to be predominantly limited by biochemistry (Acevedo-Siaca *et al*., 2020, 2021), and the same assumption might be extended to *O. glaberrima* due to a similar genomic composition (Stein *et al.*, 2018); however, we conclude from our data that stomatal limitations may be more pronounced in *O. glaberrima*.

### Accessions have adapted to variable ecological and environmental regimes in different countries

No comprehensive studies exist that tease apart the ecological and environmental variables that correlate with specific trait adaptation in *O. glaberrima*. This information is useful from an evolutionary perspective but may be essential in the selection of cultivars for abiotic stress tolerance varieties and trait-related genetic characterization.

Of note, we identified a significant association between the climate PC4 and phenotype PC1 (Fig. 8D; File S12 at Zenodo). This relationship suggests that key photosynthetic traits contributing to PC1 have adapted in response from climatic pressures associated with PC4, such as elevation and the combined effect of temperature and precipitation. However, these are broad observations for climatic–trait correlations across the African continent, lacking resolution that can be seen in studies on a discrete geographical area (Wolfe and Tonsor, 2014).

For the selection of abiotic stress tolerance-adapted cultivars, the H-clustering analyses would be of particular use, as this generated three distinct clades of *O. glaberrima* accessions stemming from similar climatic and phenotypic variables. Furthermore, the climatic H-clustering demonstrated clear grouping of accessions due to country of origin (Fig. 7B), suggesting that a higher resolution analysis of environmental effect on trait adaptation would be beneficial.

We identified adaptation based upon ecotype in the PCA (Fig. 8B), supporting a known distinction between *O. glaberrima* upland and lowland phenotypes (Ghesquière, 1997). However, there is no comprehensive description in the literature of the physiological differences that contribute to these ecotypes. Though due to the unequal representation of accessions from each ecological niche in this analysis, it is difficult to obtain a clear indication of the effect of ecotype on trait adaptation.

The environmental analysis completed here produces useful information of accessions displaying similar phenotypic qualities because of environmental adaptation. Equally, this also highlights the requirement for a dedicated study to truly elucidate the environmental and ecological trait adaptation of *O. glaberrima*, utilizing equally represented accessions from a range of ecotypes and assessing physiological adaptation to climatic variables at a range of spatial scales.

## Conclusions

Here, we have demonstrated that *O. glaberrima* has broad, heritable natural variation in a range of important traits, which are likely to aid in the improvement of *O. sativa*. This is the first study to describe photosynthetic, photoprotection, and dynamic traits in *O. glaberrima*, the size of which is not matched in panels of *O. sativa* accessions. The phenotyping efforts compiled here will provide a basis for the identification of interesting traits for physiology research, aid in the selection of accessions for crop improvement efforts, and provide information for genetic characterization.

## Supplementary data

The following supplementary data are available at *JXB* online. 

Fig. S1. Original, un-fitted data for induction and reduction of CO_2_ assimilation (A), stomatal conductance (gs) and NPQ vs time.

Fig. S2. Correlations between the best linear unbiased predictor (BLUP) values and the original mean.

Fig. S3. Correlation matrix of all phenotypic traits measured.

Fig. S4. Modelled curves for two extreme *O. glaberrima* accessions and *O. sativa* IR64, plotted on a log scale.

Fig. S5. Linear regression plots showing strong positive correlations between the actual measurement vs modelled estimate values.

Fig. S6. Plots showing the scree plot and trait loadings for the phenotypic data PCA analysis.

Fig. S7. H-clustering dendrogram of 105 *O. glaberrima* accessions and frequency plots generated from the H-clustering analysis of the phenotypic data from 155 *O. glaberrima* accessions.

Fig. S8. Correlation matrix for all phenotypic and climatic data, alongside their principal components.

Table S1. List of parameter abbreviations, definitions and units of measurement.

Table S2. List of *O. glaberrima* ID codes, country of origin and ecology.

Table S3. Estimated model outputs.

erab459_suppl_Supplementary_Figures

erab459_suppl_Supplementary_Tables

## Data Availability

Data supporting the findings of this study are available within the paper and within its supplementary data published online. The data Files (S1–S12) are deposited at the Zenodo repository: https://doi.org/10.5281/zenodo.5555930; Murchie, 2021. Any other data are available from the corresponding author upon request.
