# Supplementary material for: Out of Africa: characterizing the natural variation in dynamic photosynthetic traits in a diverse population of African rice (Oryza glaberrima)
Source: J Exp Bot. 2021 Oct 17;73(10):3283–98. doi: 10.1093/jxb/erab459 (PMC9126740; doi:10.1093/jxb/erab459)

List of Supplementary Figures and Tables

Supplementary Figure Legends

Supp. Figure 1: Original, un-fitted data for *A*, *gs* and NPQ vs time. Gas exchange data was logged every 10 s. (a) Mean values for *A* (mmol m^{-2} s^{-1}); (b) individual replicates for *A*; (c) Mean values for *gs* (mmol m^{-2} s^{-1}); (d) individual replicates for *gs*; **(e)** Mean values for NPQ; (f) individual replicates for NPQ.

Supp. Figure 2: Correlations between the best linear unbiased predictor (BLUP) values and the original mean. The BLUP value is generated from the linear mixed effect model that was used to account for undesirable variation due to spatial and temporal effects on the phenotype. The BLUP is then used to calculate the adjusted mean value. Here we grouped the plots into trait types: (a) steady state gas exchange and chlorophyll fluorescence; (b) dynamic CO_2 assimilation; (c) dynamic stomatal conductance; (d) dynamic NPQ; (e) plant morphology.

Supp. Figure 3: Correlation matrix of all phenotypic traits measured. *R* value is indicated by colour, shown in the right-hand scale bar. Stars show the significance between traits; p<0.001***, p<0.01**, p<0.05*.

Supp. Figure 4: Modelled curves for two extreme *O. glaberrima* accessions and *O. sativa* IR64, plotted on a log scale, for example *A*, *gs* and NPQ induction and relaxation dynamic traits; **(**a) $A_{\text{i rate}}$, (b) $A_{\text{r slope}}$, (c) $gs_{\text{i rate}}$, (d) $gs_{\text{r slope}}$, (e) $\text{NPQ}_{\text{i slope}}$ and (f) $\text{NPQ}_{\text{r slope}}$. The lines on all plots from the y and x-axes to the curve show the time to reach 50% of the curve maximum.

Supp. Figure 5: Linear regression plots showing strong positive correlations between the actual measurement vs modelled estimate values for (a) A_{max} vs $A_{\text{i max}}$, (b) gs_{max} vs $gs_{\text{i max}}$ and (c) NPQ_{max} vs $\text{NPQ}_{\text{i max}}$.

Supp. Figure 6: Plots showing the (a) scree plot and (b) trait loadings for the phenotypic data PCA analysis.

Supp. Figure 7: (a) H-clustering dendrogram of 105 *O. glaberrima* accessions analysed for similarities based on climatic traits, showing 3 distinct clusters. Frequency plots generated from the H-clustering analysis of the phenotypic data from 155 *O. glaberrima* accessions, showing the frequency of accessions in each cluster for (b) ecological niche and (c) country of origin.

Supp. Figure 8: Correlation matrix for all phenotypic and climatic data, alongside with their principal components. Correlation strength is indicated by colour, shown in the right-hand scale bar. Stars show the significance between traits; p<0.001***, p<0.01**, p<0.05*.

Supplementary Tables:

Supplementary Table 1: List of parameter abbreviations, definitions and units of measurement.

Supplementary Table 2: List of *O. glaberrima* ID codes, country of origin and ecology.

Supplementary Table 3a: Estimated LL.4 model outputs on carbon assimilation (*A*) IRGA induction data, showing the 4 replicates for accession IRGC_96726.

Supp. Figure 1a.

Mean CO_2 assimilation vs time for each accession.

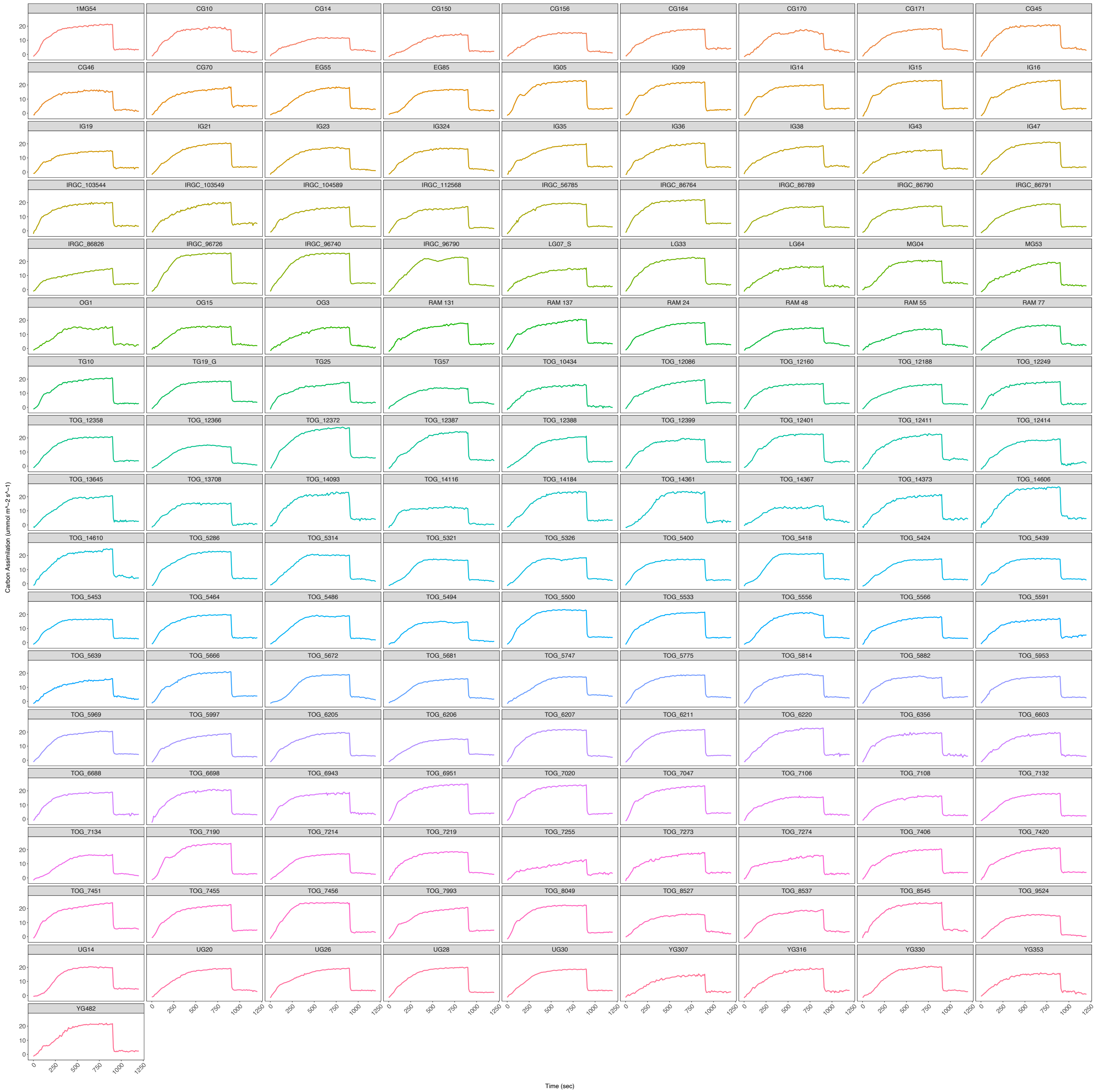

Supp. Figure 1b.

Individual replicate CO_2 assimilation vs time for each accession.

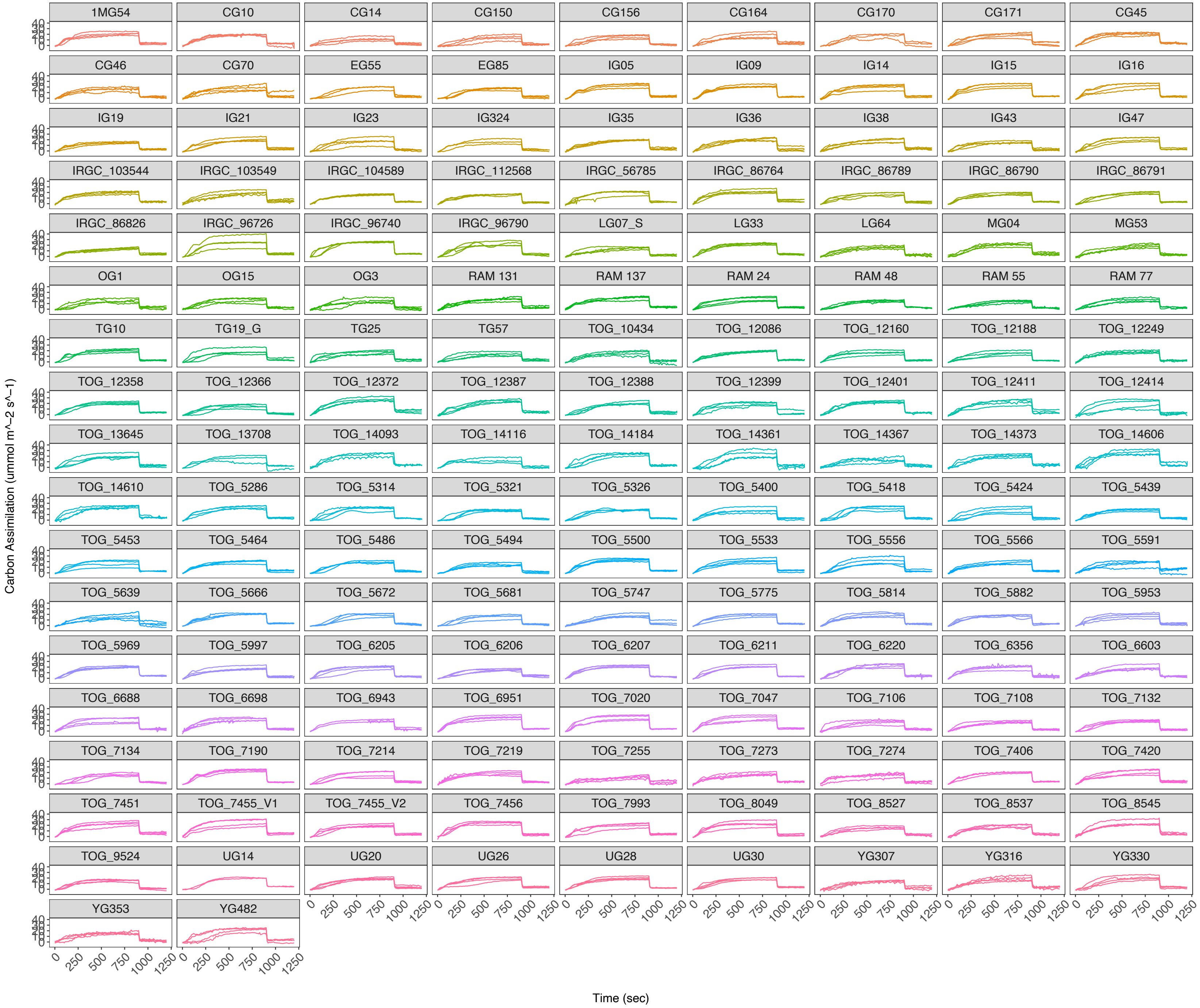

Supp. Figure 1c.

Mean stomatal conductance vs time for each accession.

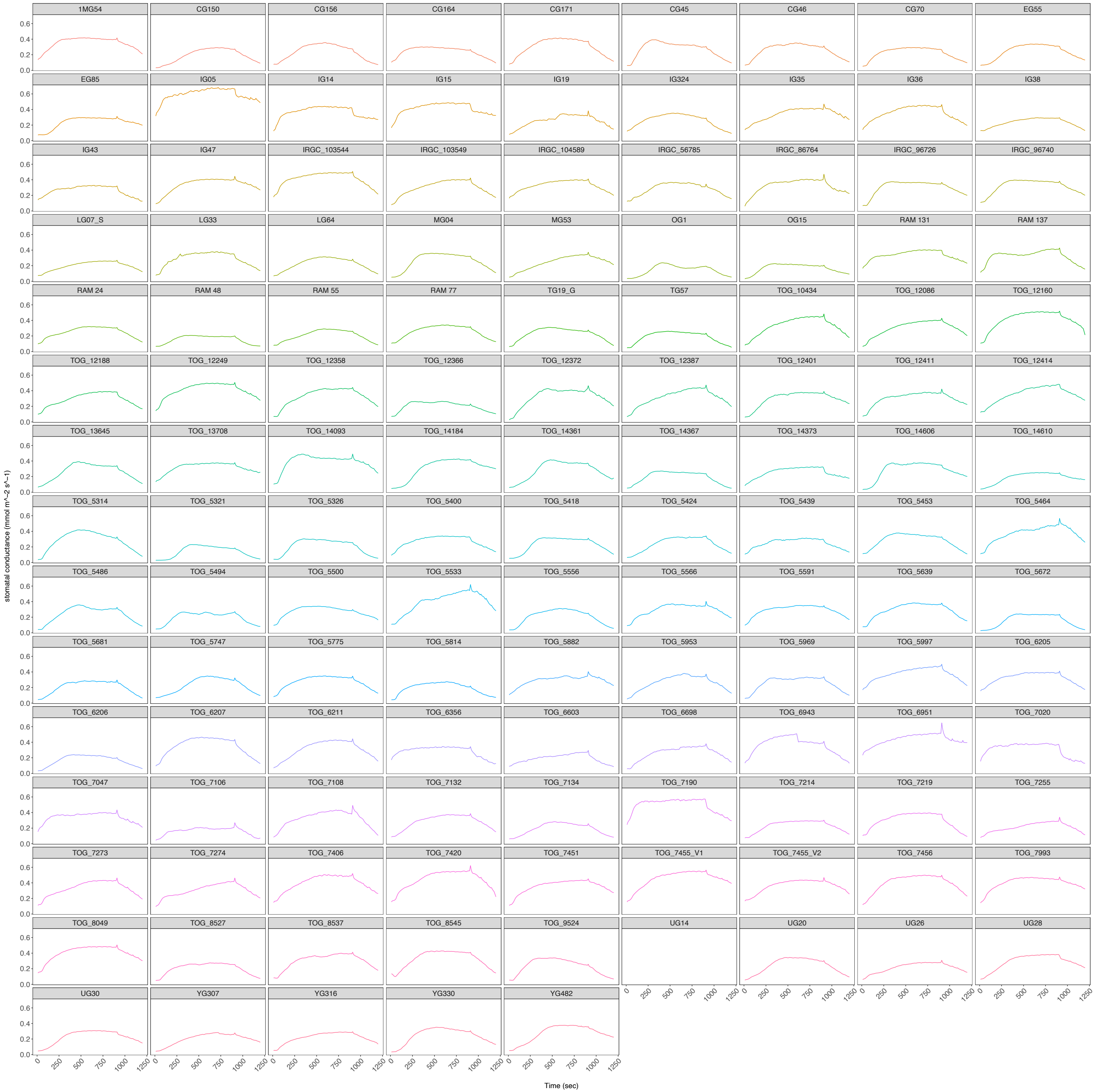

Supp. Figure 1d.

Individual replicate stomatal conductance vs time for each accession.

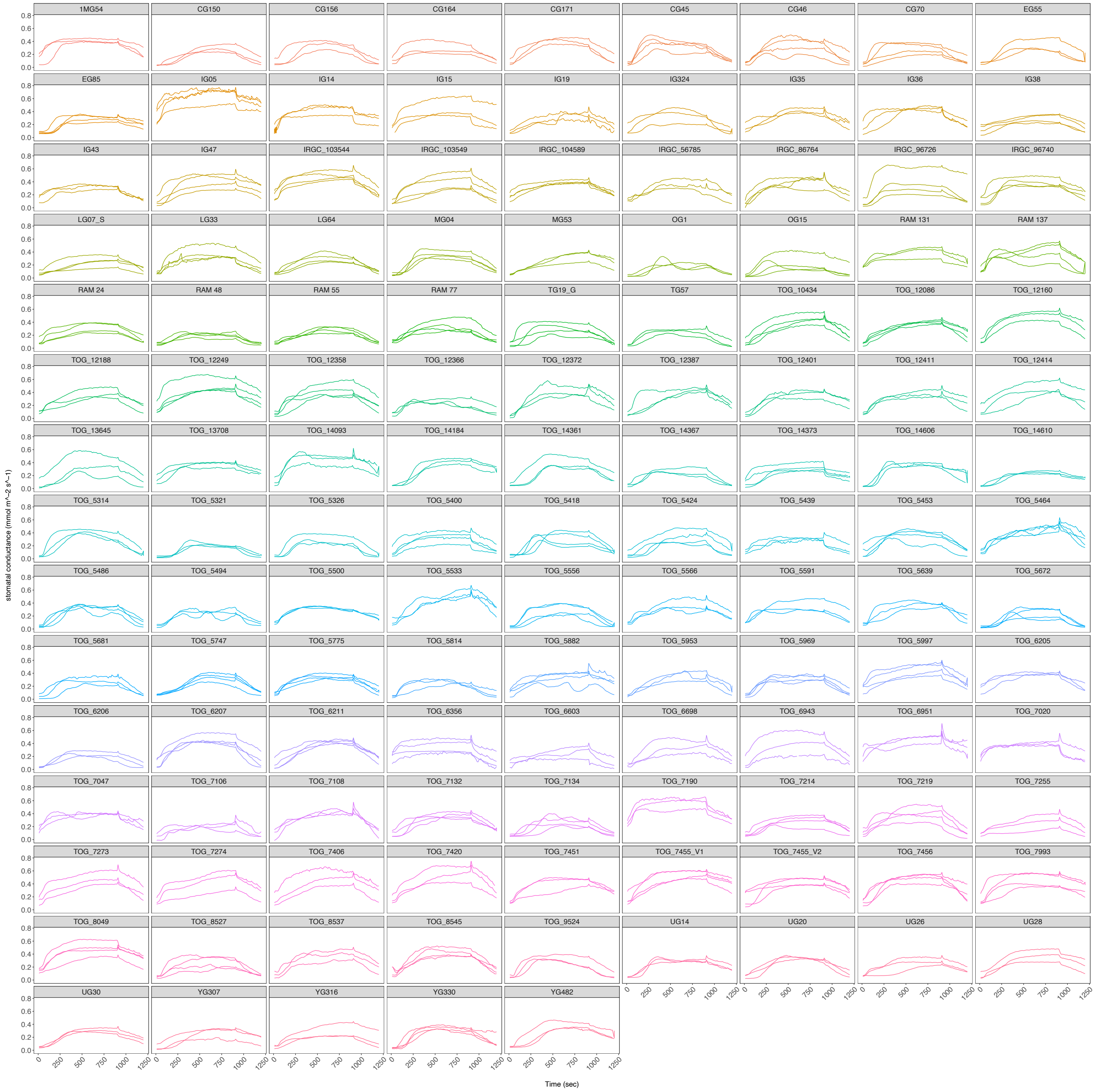

Supp. Figure 1e.

Mean NPQ vs time for each accession.

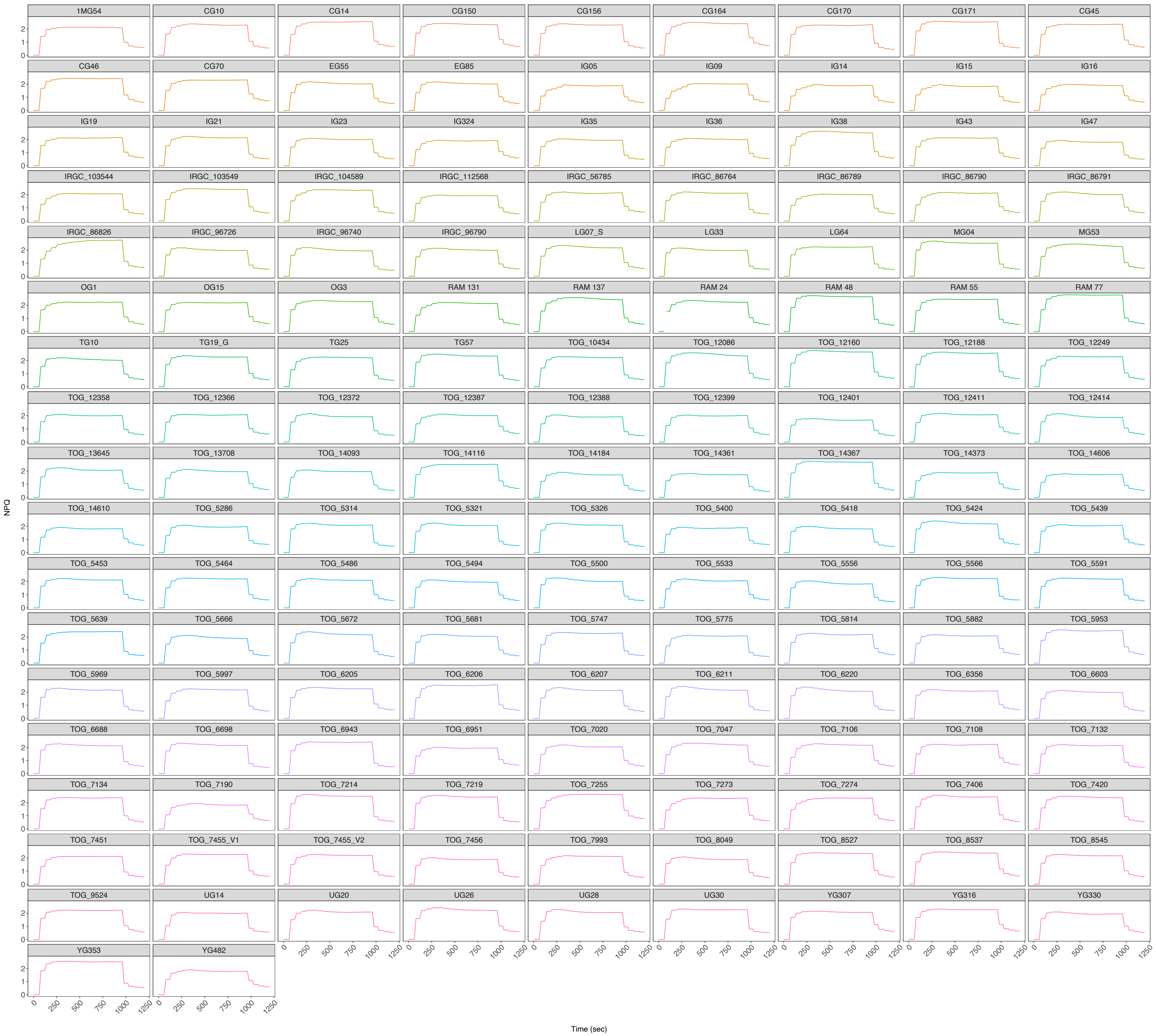

Supp. Figure 1f.

Individual replicate NPQ vs time for each accession.

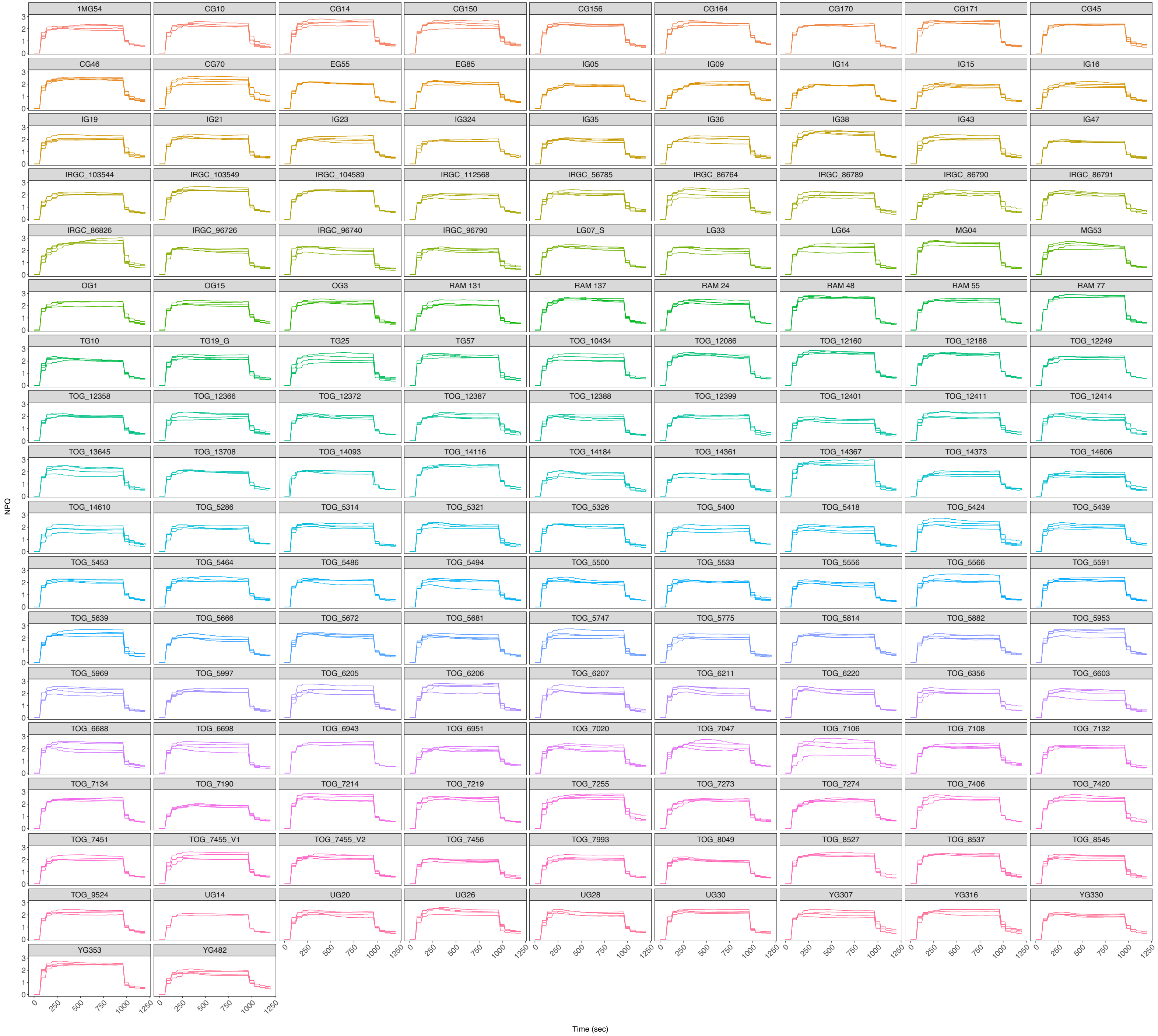

Supp. Figure 2a

Steady state gas exchange and chlorophyll fluorescence traits

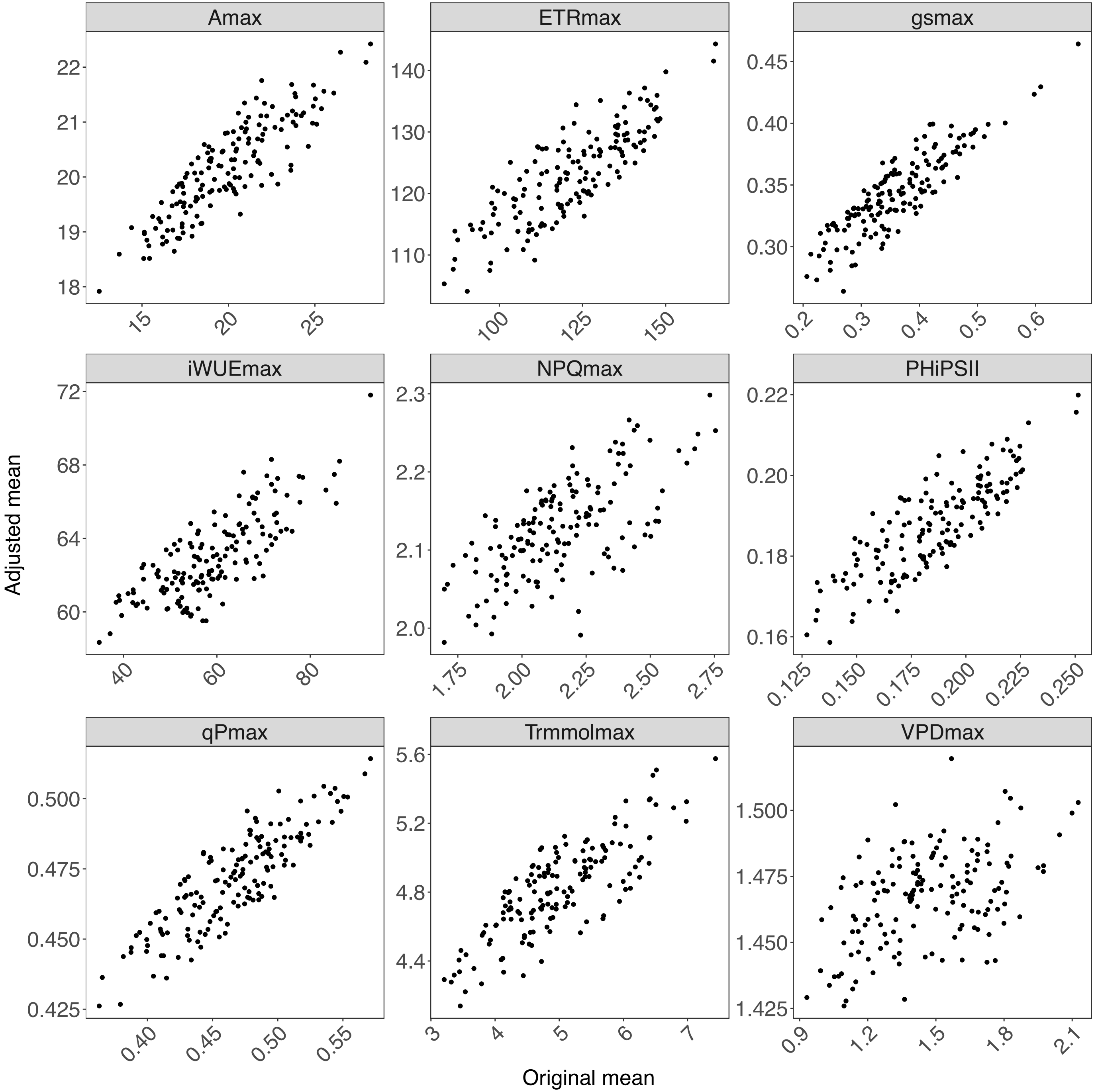

Supp. Figure 2b

Dynamic CO2 assimilation

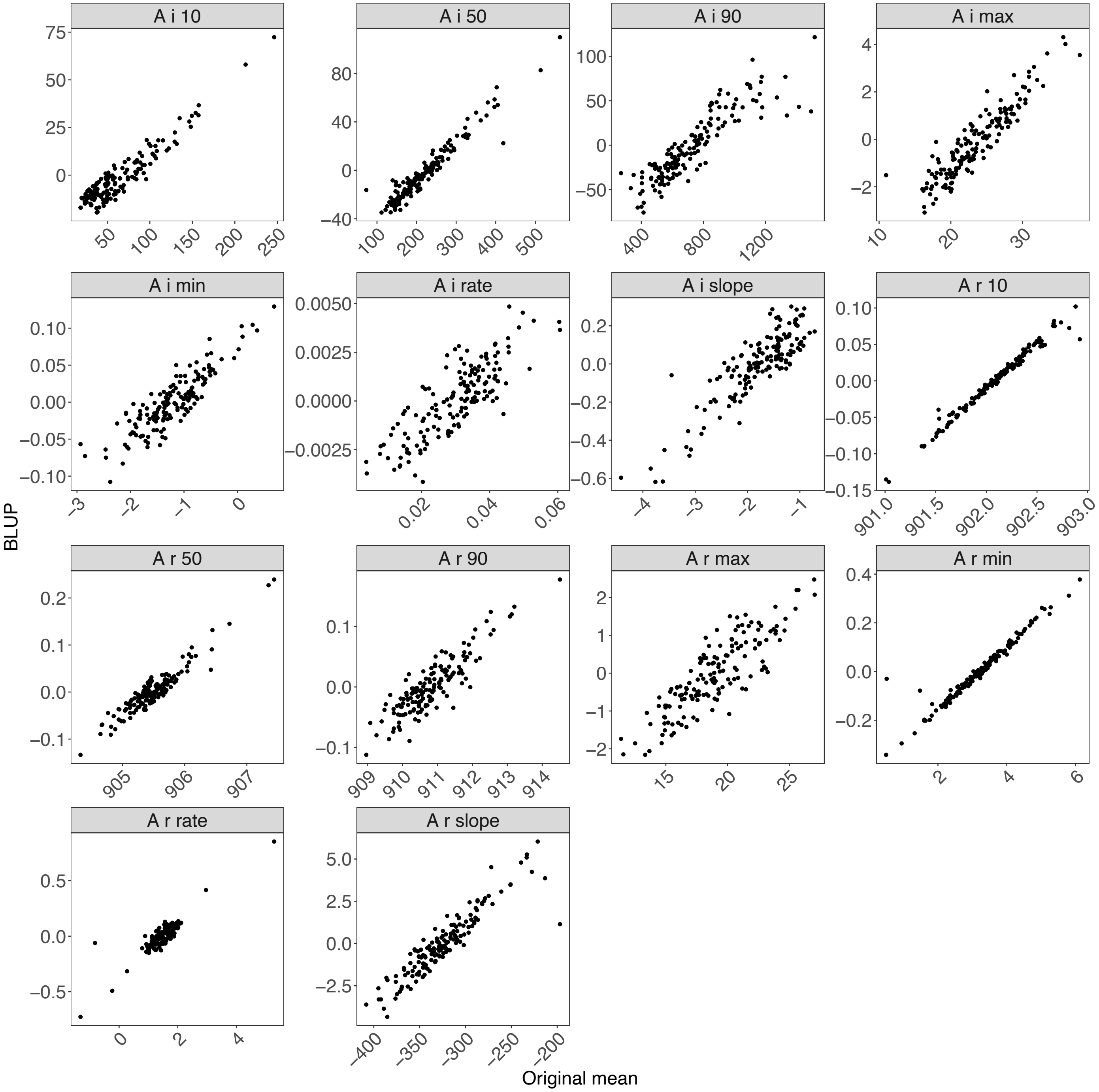

Supp. Figure 2c

Dynamic stomatal conductance traits

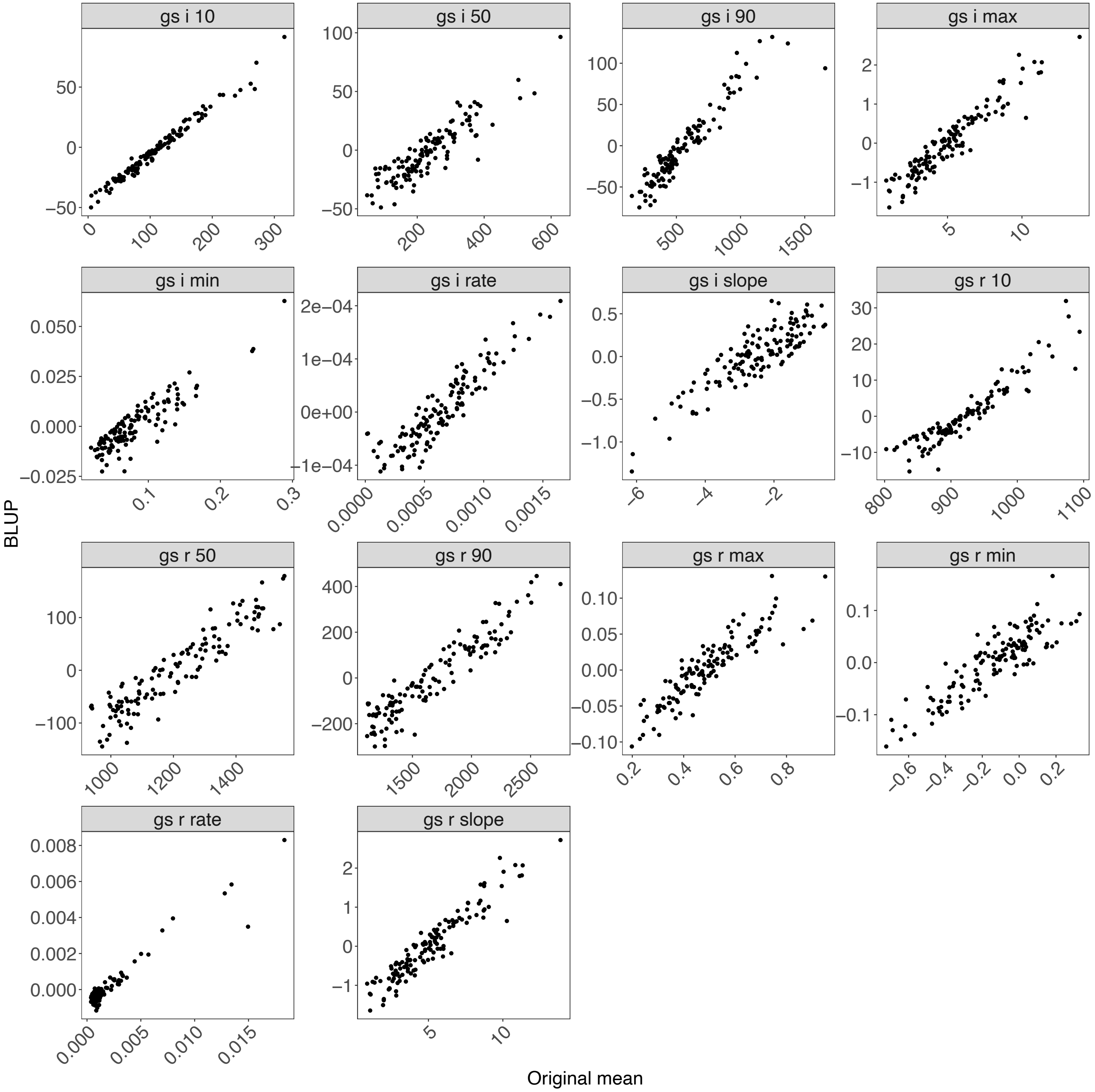

Supp. Figure 2d
Dynamic NPQ traits

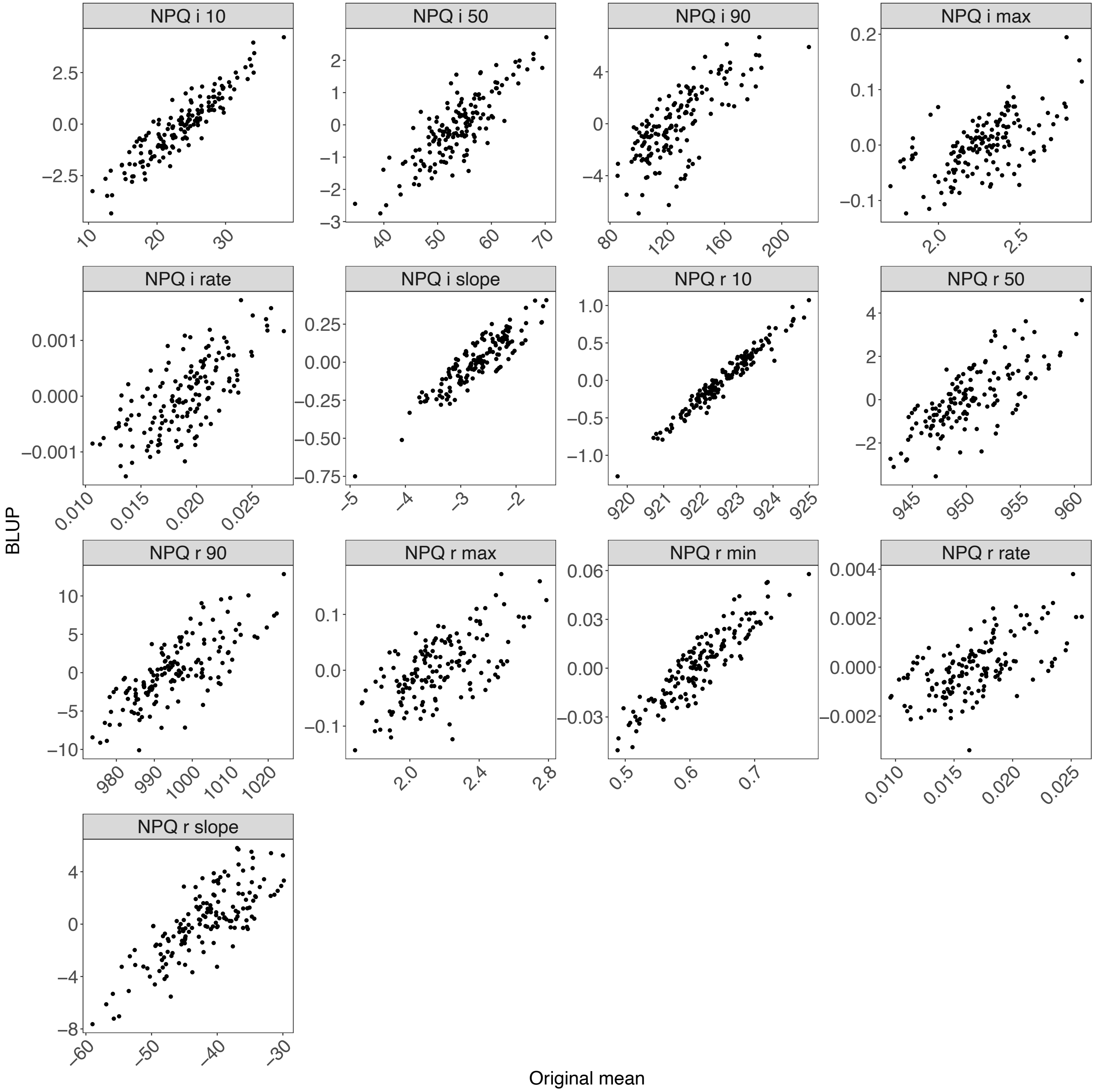

Supp. Figure 2e

Morphology traits

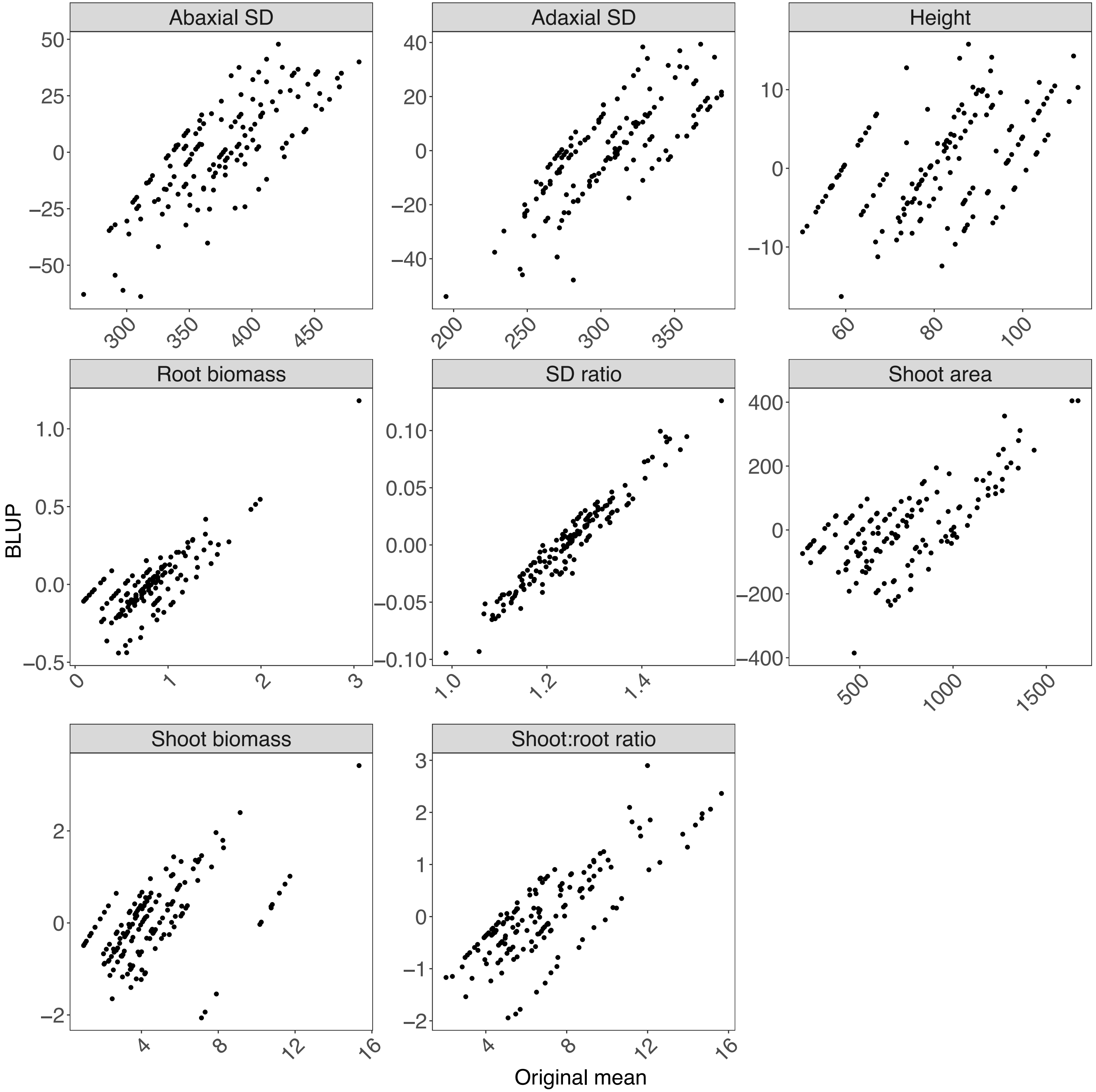

Supp. Figure 3

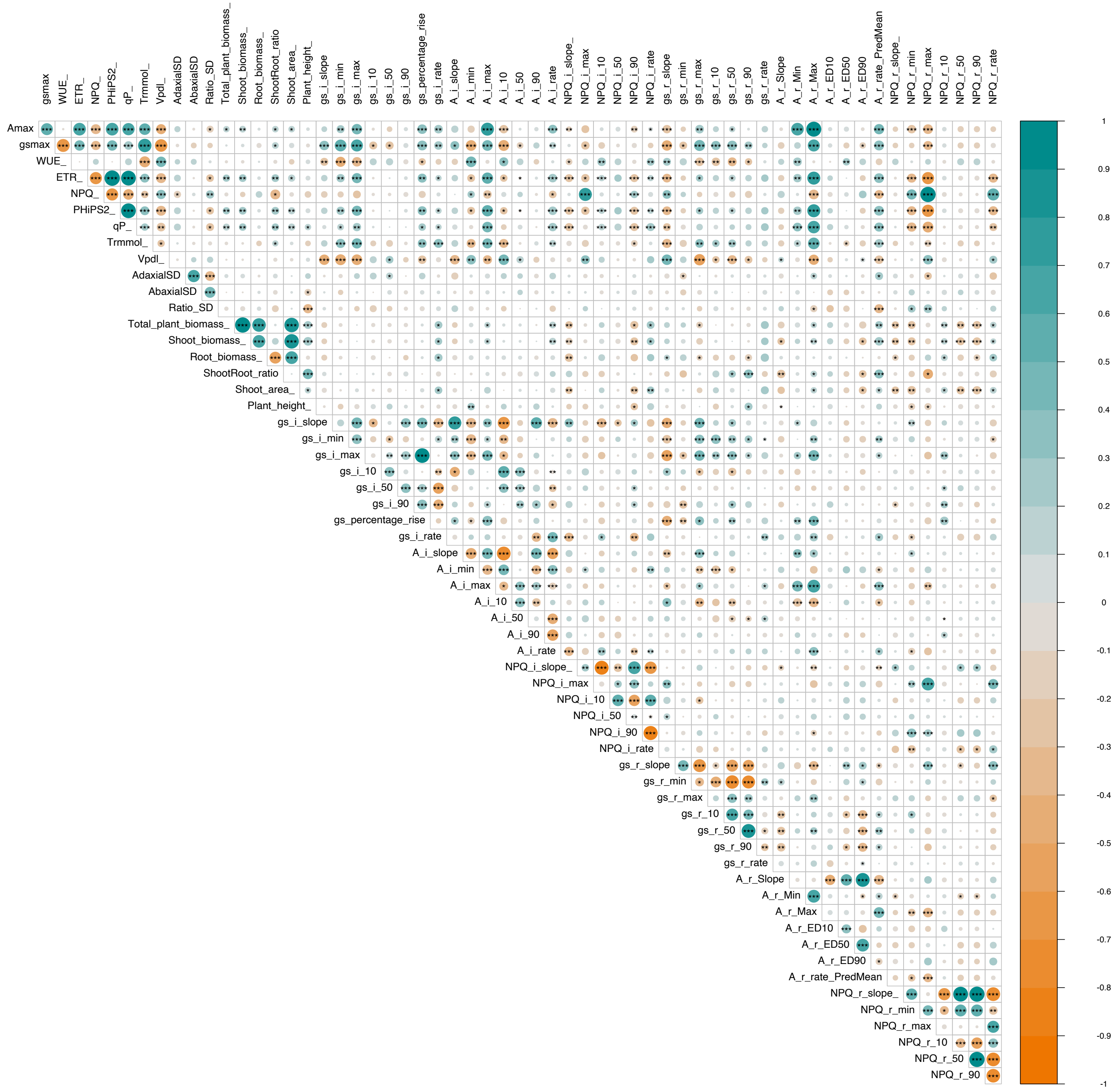

Supp. Figure 4

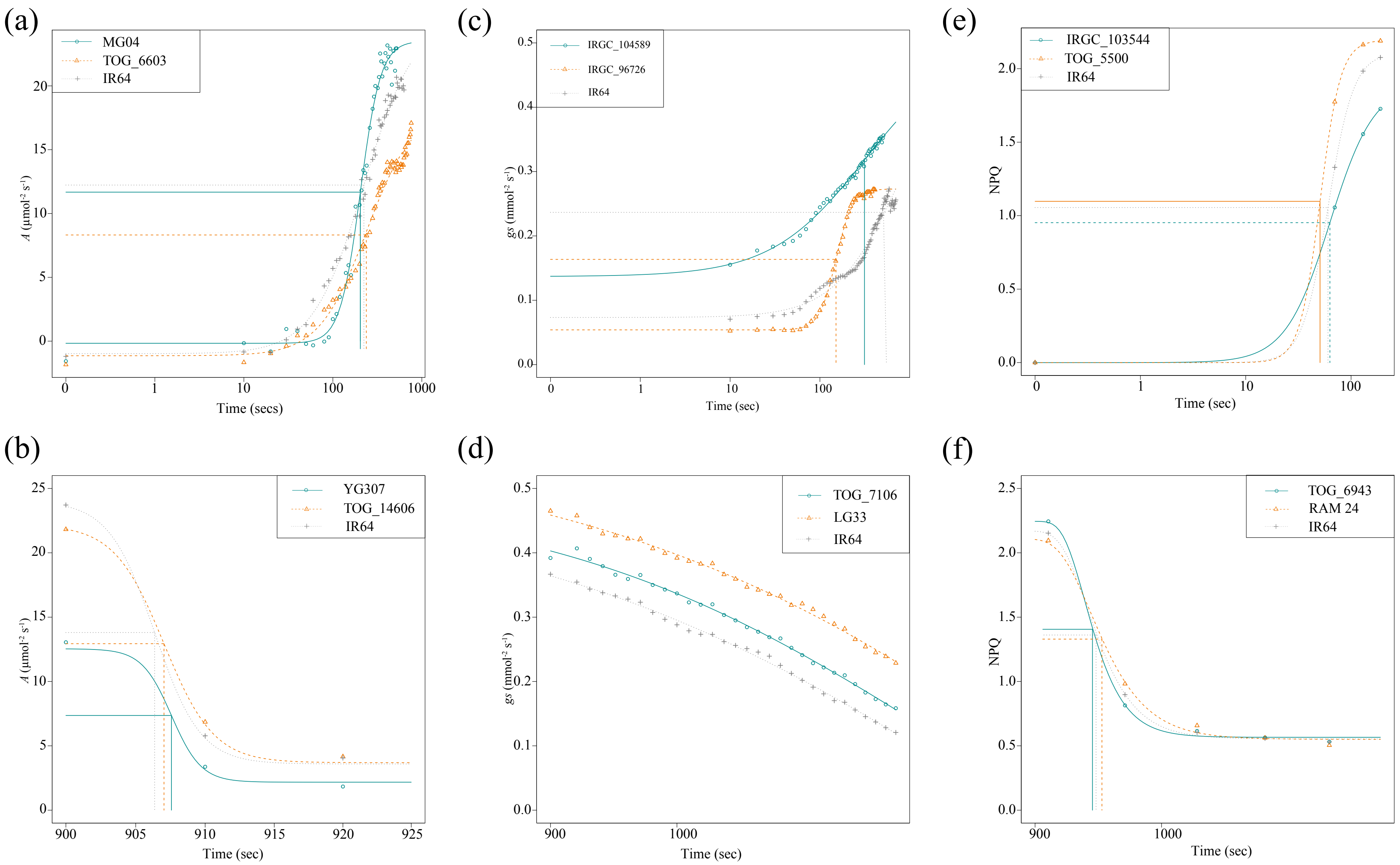

Supp. Figure 5

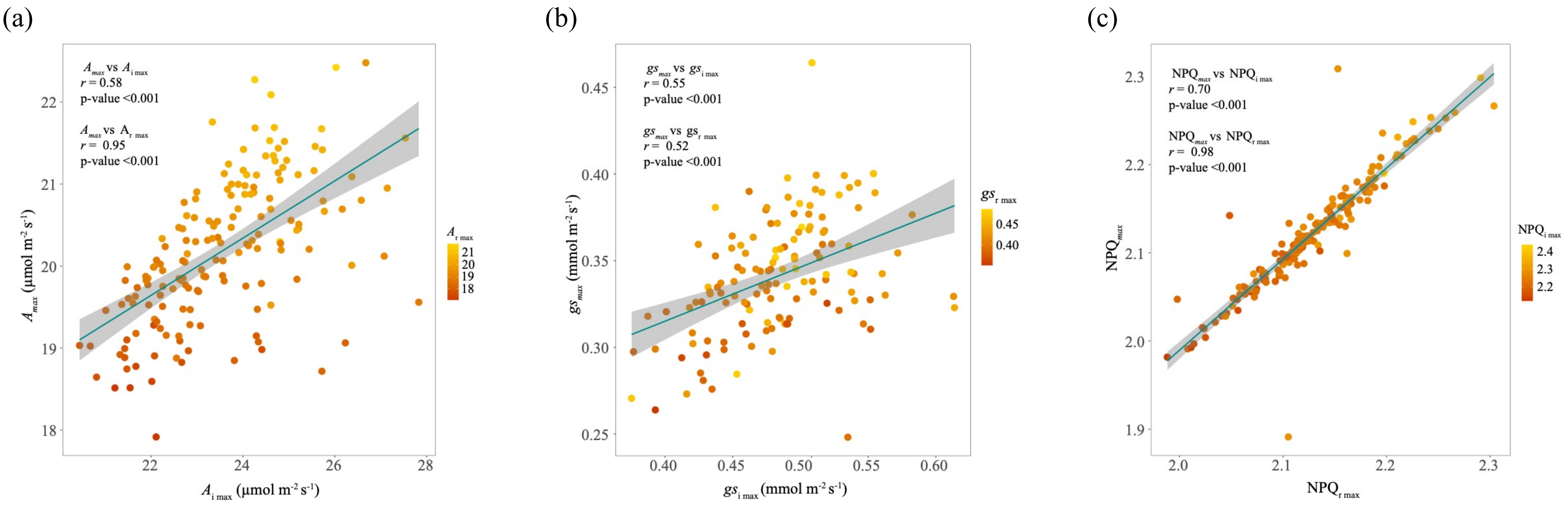

Supp. Figure 6

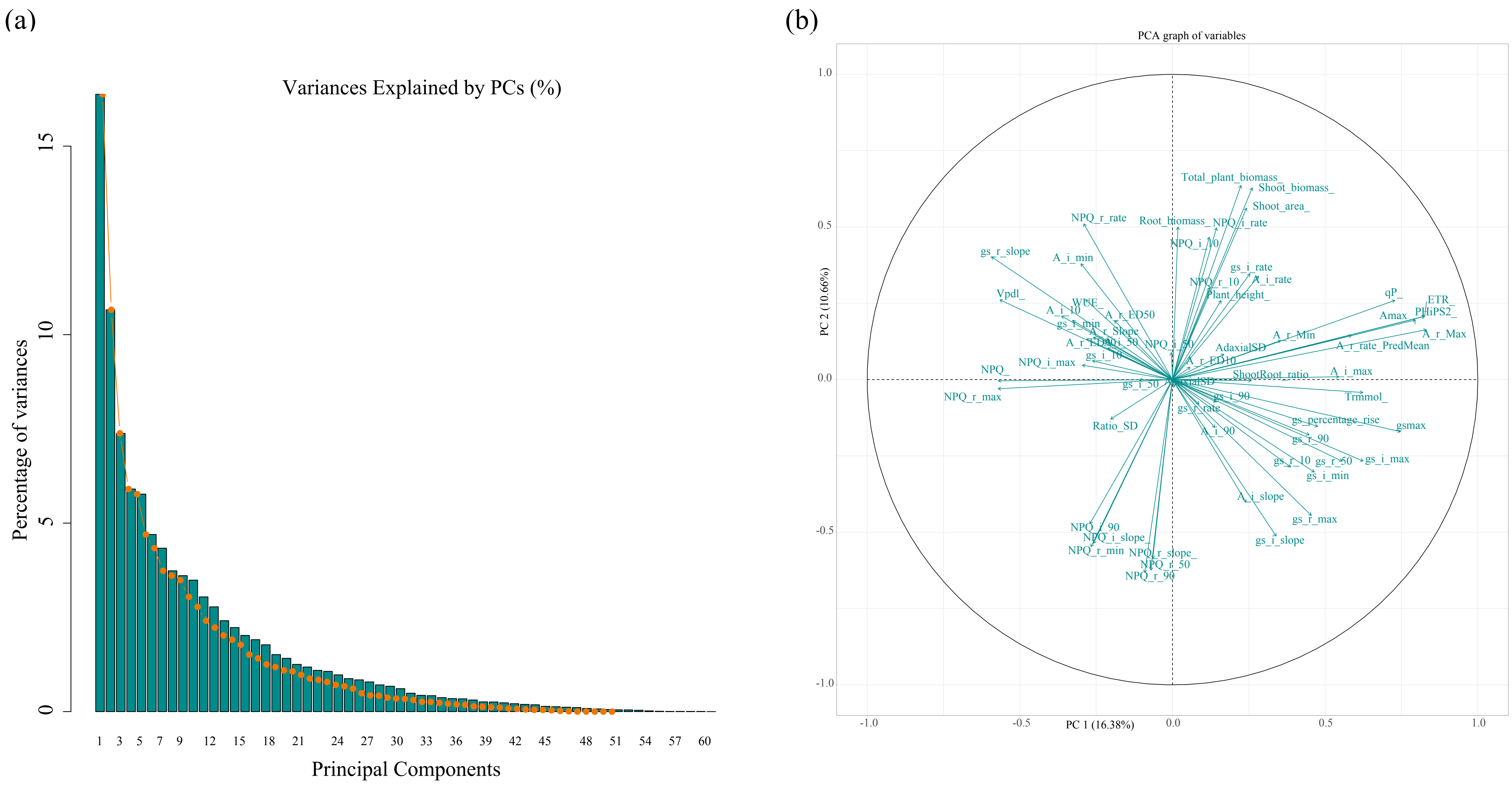

Supp. Figure 7

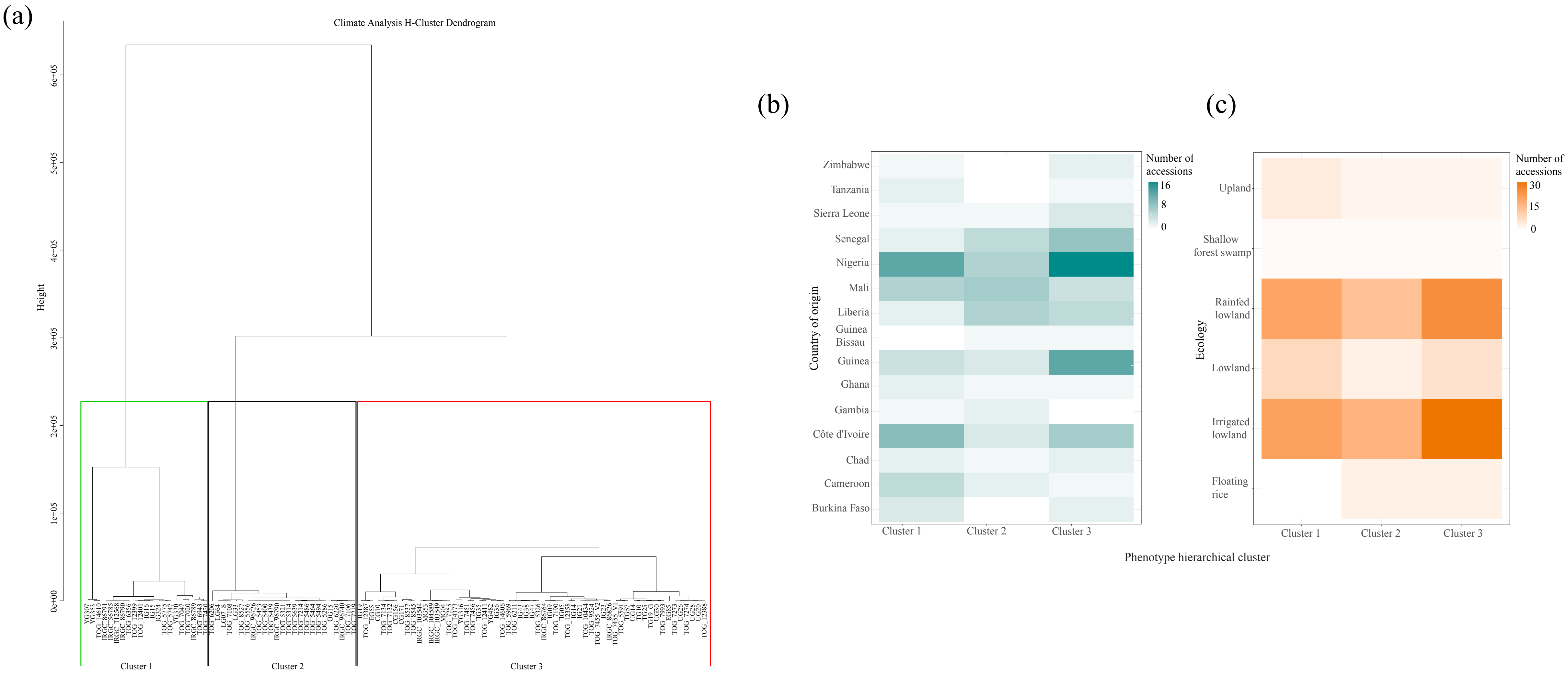

Supp. Figure 8

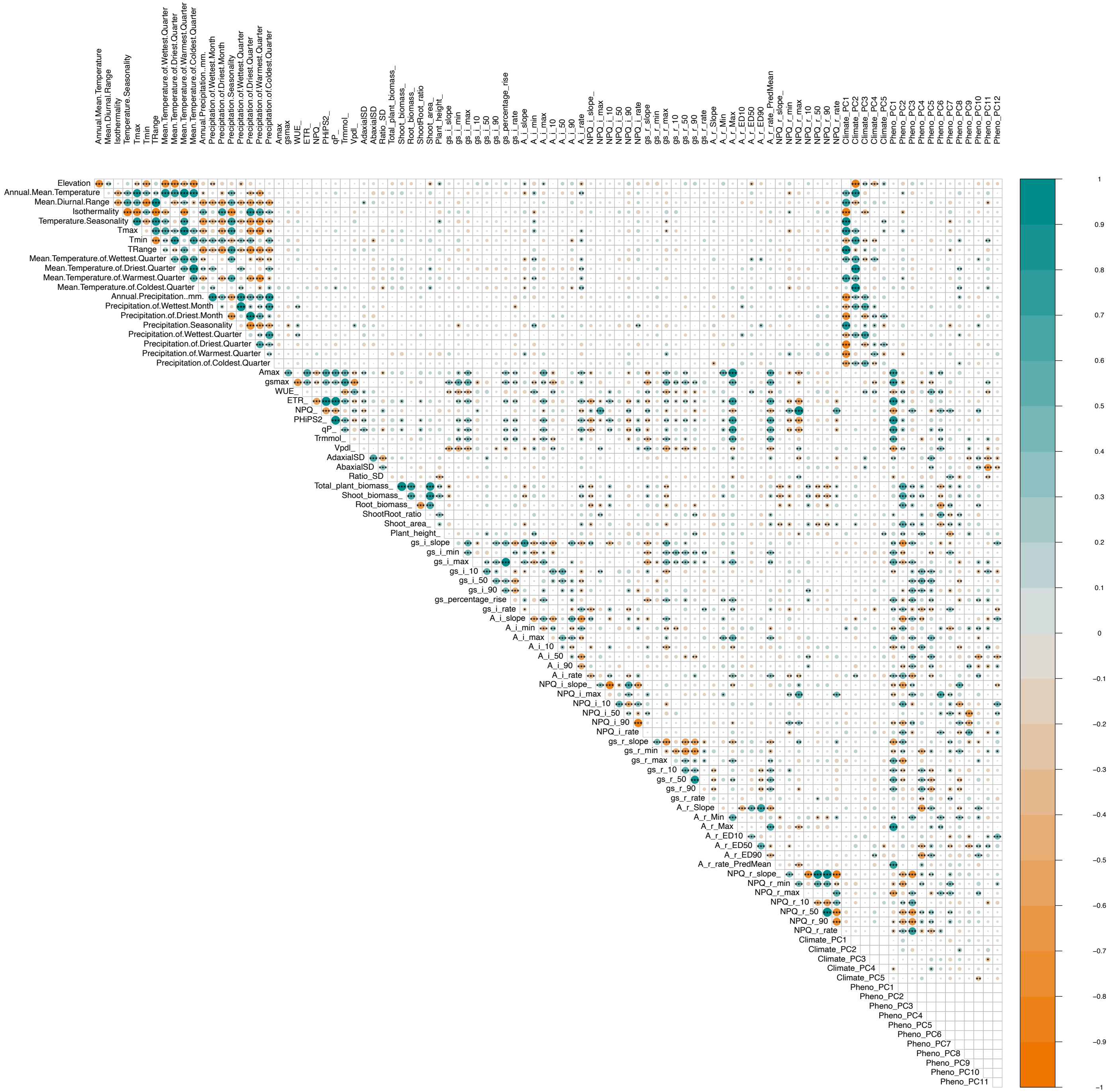

Supplement: erab459_suppl_Supplementary_Figures [file erab459_suppl_supplementary_figures.pdf]
