# Supplementary material for: Out of Africa: characterizing the natural variation in dynamic photosynthetic traits in a diverse population of African rice (Oryza glaberrima)
Source: J Exp Bot. 2021 Oct 17;73(10):3283–98. doi: 10.1093/jxb/erab459 (PMC9126740; doi:10.1093/jxb/erab459)
Supplement: erab459_suppl_Supplementary_Tables [file erab459_suppl_supplementary_tables.pdf]

Supplementary Tables

Supplementary Table 1: List of parameter abbreviations, definitions and units of measurement.

Abbreviation	Definition	Units
PPFD	Photosynthetically active photon flux density	µmol m^{-2} s^{-1}
A	Net CO_2 assimilation	µmol m^{-2} s^{-1}
g_s	Stomatal conductance to water vapour	mmol m^{-2} s^{-1}
iWUE	Intrinsic water use efficiency.	mmol mol^{-1}
ETR	Electron transport rate of PSII.	µmol electrons m^{-2} s^{-1}
NPQ	Non-photochemical quenching.	
ϕPSII	Relative quantum yield of photochemical energy conversion at steady state *A*.	
VPD	Vapour pressure difference between leaf and air.	kPa
Trmmol	Transpiration rate.	mmol H_2O m^{-2} s^{-1}
qP	Photochemical quenching	
X trait $_{max}$	Maximum of X trait achieved under 1500 mmol m^{-2} s^{-1} PPFD.	Unit of X trait
SD	Stomatal density	mm^{-2}
Abaxial SD	SD on the lower leaf side	mm^{-2}
Adaxial SD	SD on the upper leaf side	mm^{-2}
A_i | gs_i | NPQ_i	Induction response to a change of PPFD from 0 to 1500 mmol m^{-2} s^{-1} (difference between)	A_i = µmol m^{-2} s^{-1}<br>gs_i = mmol m^{-2} s^{-1}
gs_r | A_r | NPQ_r	Relaxation response to a change of PPFD from 1500 to 100 mmol m^{-2} s^{-1}	A_r = µmol m^{-2} s^{-1}<br>gs_r = mmol m^{-2} s^{-1}

$gs_{i\ slope}$ | $A_{i\ slope}$ | $NPQ_{i\ slope}$<br>$gs_{r\ slope}$ | $A_{r\ slope}$ | $NPQ_{r\ slope}$	Model estimated slope gradient for the induction and relaxation response curves.	
$gs_{i\ min}$ | $A_{i\ min}$<br>$gs_{r\ min}$ | $NPQ_{r\ min}$	Model estimated minimum value at the beginning of an induction curve and at the end of a relaxation curve.	A_i | A_r = µmol m^{-2} s^{-1}<br>gs_i | gs_r = mmol m^{-2} s^{-1}
$gs_{i\ max}$ | $A_{i\ max}$ | $NPQ_{i\ max}$<br>$gs_{r\ max}$ | $A_{r\ max}$ | $NPQ_{r\ max}$	Model estimated maximum value at the top of an induction curve, for A_i and gs_i, or a relaxation curve, for gs_r.	A_i | A_r = µmol m^{-2} s^{-1}<br>gs_i | gs_r = mmol m^{-2} s^{-1}
$gs_{i\ 10}$ | $A_{i\ 10}$ | $NPQ_{i\ 10}$<br>$gs_{r\ 10}$ | $A_{r\ 10}$ | $NPQ_{r\ 10}$	Model estimated time taken to reach 10% of the maximum value achieved on the response curve.	Time (secs)
$gs_{i\ 50}$ | $A_{i\ 50}$ | $NPQ_{i\ 50}$<br>$gs_{r\ 50}$ | $A_{r\ 50}$ | $NPQ_{r\ 50}$	Model estimated time taken to reach 50% of the maximum value achieved on the response curve.	Time (secs)
$gs_{i\ 90}$ | $A_{i\ 90}$ | $NPQ_{i\ 90}$<br>$gs_{r\ 90}$ | $A_{r\ 90}$ | $NPQ_{r\ 90}$	Model estimated time taken to reach 90% of the maximum value achieved on the response curve.	Time (secs)
$gs_{i\ rate}$ | $A_{i\ rate}$ | $NPQ_{i\ rate}$	Unit per second taken to induce from 0 seconds to the time taken to achieve 90% of the upper limit.	Trait unit per second
$gs_{r\ rate}$ | $A_{r\ rate}$ | $NPQ_{r\ rate}$	Unit per second taken to relax from 900 seconds to the time taken to achieve 90% of the lower limit.	Trait unit per second

Supplementary Table 2: List of *O. glaberrima* ID codes, country of origin and ecology.

Accession code	Ecology	Country of origin	African region
IRGC_96726	Irrigated lowland	Nigeria	West coast
TOG_5314	Irrigated lowland	Nigeria	West coast
TOG_5321	Rainfed lowland	Nigeria	West coast
TOG_5326	Shallow forest swamp	Nigeria	West coast
IRGC_96740	Irrigated lowland	Nigeria	West coast
TOG_5418	Lowland	Nigeria	West coast
TOG_5424	Rainfed lowland	Nigeria	West coast
TOG_5453	Shallow forest swamp	Nigeria	West coast
TOG_5486	Rainfed lowland	Nigeria	West coast
TOG_5494	Rainfed lowland	Nigeria	West coast
TOG_5500	Rainfed lowland	Nigeria	West coast
TOG_5556	Rainfed lowland	Nigeria	West coast
IRGC_86764	Irrigated lowland	Ghana	West coast
TOG_5666	Rainfed lowland	Nigeria	West coast
TOG_5672	Rainfed lowland	Nigeria	West coast
IRGC_96790	Irrigated lowland	Nigeria	West coast
TOG_5681	Rainfed lowland	Nigeria	West coast
TOG_5814	Rainfed lowland	Liberia	West coast
IRGC_56785	Irrigated lowland	Liberia	West coast
TOG_5882	Rainfed lowland	Nigeria	West coast
IRGC_112568	Irrigated lowland	Liberia	West coast
IRGC_86789	Irrigated lowland	Liberia	West coast
IRGC_86790	Irrigated lowland	Liberia	West coast
IRGC_86791	Irrigated lowland	Liberia	West coast
TOG_5953	Rainfed lowland	Nigeria	West coast
TOG_5969	Irrigated lowland	Nigeria	West coast
TOG_6205	Irrigated lowland	Guinea	West coast
TOG_6206	Irrigated lowland	Zimbabwe	South inland
TOG_6207	Irrigated lowland	Zimbabwe	South inland

TOG_6211	Irrigated lowland	Nigeria	West coast
TOG_6220	Irrigated lowland	Burkina Faso	West inland
TOG_6356	Rainfed lowland	Liberia	West coast
TOG_6603	Rainfed lowland	Liberia	West coast
TOG_6688	Rainfed lowland	Liberia	West coast
TOG_6698	Rainfed lowland	Liberia	West coast
TOG_6943	Irrigated lowland	Sierra Leone	West coast
TOG_6951	Irrigated lowland	Sierra Leone	West coast
TOG_7020	Irrigated lowland	Sierra Leone	West coast
TOG_7047	Irrigated lowland	Sierra Leone	West coast
TOG_7106	Irrigated lowland	Mali	West inland
TOG_7108	Irrigated lowland	Mali	West inland
TOG_5286	Rainfed lowland	Nigeria	West coast
TOG_5400	Lowland	Nigeria	West coast
TOG_5439	Rainfed lowland	Nigeria	West coast
LG33	Lowland	Mali	West inland
TOG_5464	Rainfed lowland	Nigeria	West coast
TOG_5533	Lowland	Nigeria	West coast
TOG_5566	Rainfed lowland	Nigeria	West coast
TOG_5591	Rainfed lowland	Ghana	West coast
TOG_5639	Rainfed lowland	Nigeria	West coast
CG10	Irrigated lowland	Senegal	West coast
TOG_7132	Irrigated lowland	Senegal	West coast
TOG_7134	Irrigated lowland	Senegal	West coast
TOG_5747	Rainfed lowland	Liberia	West coast
TOG_5775	Rainfed lowland	Liberia	West coast
TOG_5997	Upland	Nigeria	West coast
TOG_7420	Rainfed lowland	Sierra Leone	West coast
IRGC_103544	Irrigated lowland	Mali	West inland
RAM 131	Floating Rice	Mali	West inland
RAM 137	Floating Rice	Mali	West inland
RAM 24	Floating Rice	Guinea	West coast
RAM 48	Floating Rice	Mali	West inland

RAM 55	Floating Rice	Mali	West inland
RAM 77	Floating Rice	Mali	West inland
CG14	Irrigated lowland	Senegal	West coast
IG38	Rainfed lowland	Côte d'Ivoire	West coast
TOG_14367	Irrigated lowland	Guinea	West coast
YG353	Rainfed lowland	Guinea	West coast
MG04	Lowland	Mali	West inland
TOG_7214	Irrigated lowland	Upland	West inland
CG171	Irrigated lowland	Senegal	West coast
TOG_7219	Irrigated lowland	Mali	West inland
IRGC_103549	Irrigated lowland	Mali	West inland
TOG_10434	Irrigated lowland	Côte d'Ivoire	West coast
TOG_7255	Irrigated lowland	Chad	North inland
TOG_12086	Rainfed lowland	Nigeria	West coast
TOG_12160	Rainfed lowland	Nigeria	West coast
TOG_12188	Rainfed lowland	Nigeria	West coast
TOG_12249	Rainfed lowland	Nigeria	West coast
TOG_7273	Irrigated lowland	Cameroon	West coast
TOG_7274	Shallow Forest Swamp	Cameroon	West coast
IRGC_104589	Irrigated lowland	Burkina Faso	West inland
IRGC_86826	Irrigated lowland	Ghana	West coast
TOG_7406	Rainfed lowland	Ghana	West coast
TOG_7451	Irrigated lowland	Burkina Faso	West inland
TOG_7455	Irrigated lowland	Burkina Faso	West inland
TOG_7456	Irrigated lowland	Burkina Faso	West inland
TOG_7455	Irrigated lowland	Côte d'Ivoire	West coast
TOG_7993	Irrigated lowland	Nigeria	West coast
TOG_8049	Irrigated lowland	Nigeria	West coast
TOG_8527	Irrigated lowland	Gambia	West coast
TOG_8537	Irrigated lowland	Gambia	West coast
TOG_8545	Irrigated lowland	Gambia	West coast
TOG_9524	Irrigated lowland	Côte d'Ivoire	West coast

TOG_12358	Upland	Côte d'Ivoire	West coast
TOG_12366	Rainfed lowland	Guinea-Bissau	West coast
TOG_12372	Rainfed lowland	Guinea-Bissau	West coast
TOG_12387	Rainfed lowland	Tanzania	East coast
TOG_12388	Rainfed lowland	Cameroon	West coast
TOG_12399	Upland	Guinea	West coast
TOG_12401	Upland	Guinea	West coast
TOG_12411	Upland	Guinea	West coast
TOG_12414	Upland	Guinea	West coast
YG330	Rainfed lowland	Guinea	West coast
TOG_13645	Irrigated lowland	Guinea	West coast
TOG_13708	Irrigated lowland	Guinea	West coast
TOG_14093	Irrigated lowland	Guinea	West coast
TOG_14116	Rainfed lowland	Liberia	West coast
TOG_14184	Rainfed lowland	Zimbabwe	South inland
YG482	Rainfed lowland	Guinea	West coast
TOG_14361	Rainfed lowland	Guinea	West coast
TOG_14373	Irrigated lowland	Guinea	West coast
TOG_14606	Irrigated lowland	Guinea	West coast
TOG_14610	Irrigated lowland	Guinea	West coast
TOG_7190	Irrigated lowland	Côte d'Ivoire	West coast
IG05	Rainfed lowland	Côte d'Ivoire	West coast
IG09	Rainfed lowland	Côte d'Ivoire	West coast
IG14	Rainfed lowland	Côte d'Ivoire	West coast
IG15	Rainfed lowland	Côte d'Ivoire	West coast
IG16	Rainfed lowland	Côte d'Ivoire	West coast
IG19	Rainfed lowland	Côte d'Ivoire	West coast
IG21	Rainfed lowland	Côte d'Ivoire	West coast
IG23	Rainfed lowland	Côte d'Ivoire	West coast
IG35	Rainfed lowland	Côte d'Ivoire	West coast
IG36	Upland	Côte d'Ivoire	West coast
IG43	Rainfed lowland	Côte d'Ivoire	West coast
IG47	Rainfed lowland	Côte d'Ivoire	West coast

IG324	Rainfed lowland	Côte d'Ivoire	West coast
EG55	Lowland	Tanzania	East coast
EG85	Lowland	Tanzania	East coast
UG14	Lowland	Cameroon	West coast
UG20	Lowland	Cameroon	West coast
UG26	Lowland	Cameroon	West coast
UG28	Rainfed lowland	Cameroon	West coast
UG30	Rainfed lowland	Cameroon	West coast
LG07_S	Lowland	Mali	West inland
LG64	Lowland	Mali	West inland
MG53	Lowland	Mali	West inland
1MG54	Lowland	Mali	West inland
TG10	Irrigated lowland	Chad	North inland
TG19_G	Irrigated lowland	Chad	North inland
TG25	Irrigated lowland	Chad	North inland
TG57	Irrigated lowland	Chad	North inland
CG45	Irrigated lowland	Senegal	West coast
CG46	Irrigated lowland	Senegal	West coast
CG70	Irrigated lowland	Senegal	West coast
CG150	Irrigated lowland	Senegal	West coast
CG156	Irrigated lowland	Senegal	West coast
CG164	Irrigated lowland	Senegal	West coast
CG170	Irrigated lowland	Senegal	West coast
OG1	lowland	Senegal	West coast
OG3	lowland	Senegal	West coast
OG15	lowland	Senegal	West coast
YG307	Upland	Guinea	West coast
YG316	Rainfed lowland	Guinea	West coast

Supplementary Table 3a: Estimated LL.4 model outputs on carbon assimilation (*A*) IRGA induction data, showing the 4 replicates for accession IRGC_96726.

Replicate	Parameter	Model estimate	Model SE
1	$A_{i\ min}$	-2.22	1.13
1	$A_{i\ max}$	44.07	2.10
1	$A_{i\ 50}$	147.62	10.72
1	$A_{i\ slope}$	-1.41	0.13
2	$A_{i\ min}$	-0.42	0.22
2	$A_{i\ max}$	27.43	0.86
2	$A_{i\ 50}$	177.88	5.19
2	$A_{i\ slope}$	-2.35	0.11
3	$A_{i\ min}$	-0.13	0.24
3	$A_{i\ max}$	13.39	0.37
3	$A_{i\ 50}$	175.31	4.94
3	$A_{i\ slope}$	-3.08	0.26
4	$A_{i\ min}$	-0.51	0.16
4	$A_{i\ max}$	24.12	0.24
4	$A_{i\ 50}$	151.35	1.67
4	$A_{i\ slope}$	-2.93	0.09

Supplementary Table 3b: Estimated LL.4 model outputs on stomatal conductance (*gs*) IRGA induction data, showing the 4 replicates for accession MG04.

Replicate	Parameter	Model estimate	Model SE
1	$gs_{i\ min}$	0.03	0.001
1	$gs_{i\ max}$	0.52	0.02
1	$gs_{i\ 50}$	230.95	9.97
1	$gs_{i\ slope}$	-2.15	0.08
2	$gs_{i\ min}$	0.08	0.00
2	$gs_{i\ max}$	0.35	0.00
2	$gs_{i\ 50}$	291.44	1.77
2	$gs_{i\ slope}$	-4.89	0.09
3	$gs_{i\ min}$	0.05	0.00
3	$gs_{i\ max}$	0.37	0.01
3	$gs_{i\ 50}$	241.79	4.48
3	$gs_{i\ slope}$	-3.86	0.13
4	$gs_{i\ min}$	0.04	0.00
4	$gs_{i\ max}$	0.54	0.02
4	$gs_{i\ 50}$	235.36	4.87
4	$gs_{i\ slope}$	-4.63	0.21

Supplementary Table 3c: Estimated LL.3 model outputs on non-photochemical quenching (NPQ) IRGA induction data, showing the 4 replicates for accession TOG_12188.

Replicate	Parameter	Model estimate	Model SE
1	$NPQ_{i\ max}$	2.77	0.00
1	$NPQ_{i\ 50}$	49.08	0.01
1	$NPQ_{i\ slope}$	-1.37	0.00
2	$NPQ_{i\ max}$	2.74	0.00
2	$NPQ_{i\ 50}$	54.05	0.00
2	$NPQ_{i\ slope}$	-2.55	0.00
3	$NPQ_{i\ max}$	2.67	0.00
3	$NPQ_{i\ 50}$	56.50	0.00
3	$NPQ_{i\ slope}$	-2.00	0.00
4	$NPQ_{i\ max}$	2.73	0.00
4	$NPQ_{i\ 50}$	56.9	0.00
4	$NPQ_{i\ slope}$	-2.31	0.00

Supplementary Table 3d: Estimated W2.4 model outputs on non-photochemical quenching (NPQ) IRGA relaxation data, showing the 3 replicates for accession EG55. EG55 was one of a small number of accession where only 3 replicates were measured.

Replicate	Parameter	Model estimate	Model SE
1	$NPQ_{r\ min}$	0.60	0.03
1	$NPQ_{r\ max}$	2.09	0.04
1	$NPQ_{r\ 50}$	942.71	5.21
1	$NPQ_{r\ slope}$	-39.32	7.53
2	$NPQ_{r\ min}$	0.53	0.02
2	$NPQ_{r\ max}$	2.0	0.03
2	$NPQ_{r\ 50}$	942.71	5.00
2	$NPQ_{r\ slope}$	-41.15	7.50
3	$NPQ_{r\ min}$	0.59	0.02
3	$NPQ_{r\ max}$	2.03	0.03
3	$NPQ_{r\ 50}$	941.15	5.28
3	$NPQ_{r\ slope}$	-44.06	8.28

Supplementary Table 3e: Estimated LL.4 model outputs on stomatal conductance (*gs*) IRGA relaxation data, showing the 4 replicates for accession TOG_5326.

Replicate	Parameter	Model estimate	Model SE
1	$gs_{r\ min}$	0.05	0.00
1	$gs_{r\ max}$	3.04	0.00
1	$gs_{r\ 50}$	1000.20	3.73
1	$gs_{r\ slope}$	18.39	1.23
2	$gs_{r\ min}$	0.037	0.00
2	$gs_{r\ max}$	0.21	0.00
2	$gs_{r\ 50}$	994.68	4.37
2	$gs_{r\ slope}$	17.89	1.24
3	$gs_{r\ min}$	0.05	0.00
3	$gs_{r\ max}$	0.35	0.00
3	$gs_{r\ 50}$	1063.03	2.12
3	$gs_{r\ slope}$	15.09	0.63
4	$gs_{r\ min}$	0.03	0.00
4	$gs_{r\ max}$	0.24	0.00
4	$gs_{r\ 50}$	978.00	2.09
4	$gs_{r\ slope}$	32.02	1.60

Supplementary Table 3f: Estimated LL.4 model outputs on carbon assimilation (*A*) IRGA relaxation data, showing the 4 replicates for accession UG26.

Replicate	Parameter	Model estimate	Model SE
1	$A_{r\ min}$	5.07	0.06
1	$A_{r\ max}$	21.87	0.18
1	$A_{r\ 50}$	907.31	2.55
1	$A_{r\ slope}$	502.49	221.24
2	$A_{r\ min}$	3.38	0.05
2	$A_{r\ max}$	20.94	0.13
2	$A_{r\ 50}$	911.40	2.05
2	$A_{r\ slope}$	488.49	130.49
3	$A_{r\ min}$	2.40	0.04
3	$A_{r\ max}$	18.12	0.10
3	$A_{r\ 50}$	910.03	6.06
3	$A_{r\ slope}$	801.65	598.41
4	$A_{r\ min}$	3.45	0.08
4	$A_{r\ max}$	16.19	0.21
4	$A_{r\ 50}$	911.50	1.76
4	$A_{r\ slope}$	298.68	66.33
